# Enzymatic basis of branching and extension of *O*-Man glycans for keratan sulfate biosynthesis

**DOI:** 10.1016/j.jbc.2026.111140

**Published:** 2026-01-07

**Authors:** Tomoya Itoh, Hide-Nori Tanaka, Mohit Pareek, Masamichi Nagae, Hiroshi Manya, Akemi Ido, Sushil K. Mishra, Yasuhiko Kizuka

**Affiliations:** 1 Graduate School of Natural Science and Technology, Gifu University, Gifu, Japan; 2 Institute for Glyco-Core Research (iGCORE), Gifu University, Gifu, Japan; 3 Department of Biomedical Engineering, University of Mississippi, Mississippi, USA; 4 Department of Molecular Immunology, Research Institute for Microbial Diseases, The University of Osaka, Suita, Japan; 5 Laboratory of Molecular Immunology, Immunology Frontier Research Center (IFReC), The University of Osaka, Suita, Japan; 6 Molecular Glycobiology, Research Team for Mechanism of Aging, Tokyo Metropolitan Institute for Geriatrics and Gerontology, Tokyo, Japan

**Keywords:** glycobiology, glycoprotein biosynthesis, glycosaminoglycan, glycosylation, glycosyltransferase, keratan sulfate, *O*-mannose glycan, MGAT5B (GnT-IX)

## Abstract

*O*-Mannose (Man) glycans are branched specifically in the brain by a dedicated glycosyltransferase, *N*-acetylglucosaminyltransferase IX (GnT-IX, also known as MGAT5B). Such branching of *O*-Man glycans was reported to be involved in diseases, including demyelination and glioma, but the enzymatic mechanisms by which *O*-Man glycan is specifically recognized by GnT-IX and how branched *O*-Man glycans are subsequently elongated by other enzymes in the brain have remained unclear. To shed light on these issues, we here first compared the structural model of GnT-IX complexed with its *O*-Man substrate with the crystal structure of the homologous *N*-glycan branching enzyme GnT-V (also known as MGAT5). Several residues in GnT-IX were predicted to be critical to recognition of the *O*-Man substrate, and an enzyme assay revealed that R304 in GnT-IX is crucial for the specificity toward *O*-Man glycans. We further investigated the role of *O*-Man branching for subsequent elongation in the brain and found that the level of keratan sulfate (KS) in *O*-Man glycans was significantly reduced in GnT-IX-knockout (KO) mouse brain, suggesting that *O*-Man branching promotes KS biosynthesis. Mechanistically, our enzymatic assays of the KS biosynthetic enzymes demonstrated that B4GALT1, B4GALT4, and CHST1 exhibited significantly higher activity toward branched *O*-Man glycans than toward their linear counterparts. These results imply that branching of *O*-Man glycans by GnT-IX provides the scaffold for efficient subsequent glycan elongation. Our findings deepen our understanding of the complex biosynthetic pathway of *O*-Man glycans in the brain.

Glycosylation of proteins is one of the most common posttranslational modifications (1, 2) and critically regulates protein functions in mammals (1, 2). Glycans on proteins are classified as *N*-glycans, *O*-glycans, *C*-Man, and glycosylphosphatidylinositol, based on the amino acids to which glycans are attached, and *O*-glycans are linked to a Ser or Thr residue (2). Among *O*-glycans, *O*-Man glycans are linked to a polypeptide *via* the reducing end Man and have been demonstrated to exert essential functions in the brain and muscle. In the brain, one-third of *O*-glycans is estimated to be *O*-Man type (3), and alterations in *O*-Man glycan structure have been reported to be involved in various neural disorders, including demyelination and glioma (4, 5), suggesting the biological significance of *O*-Man glycans in the brain. Furthermore, genetic defects in the biosynthesis of a specific epitope in *O*-Man glycan designated as matriglycan result in severe muscular dystrophy with anomalies in brain structure, known as α-dystroglycanopathy (6, 7). These findings underscore the importance of understanding the regulatory mechanisms by which complex *O*-man glycans are shaped *in vivo*.

*O*-Man glycans have three types of core structures: GlcNAcβ1-2Man (core M1), GlcNAcβ1-2(GlcNAcβ1-6)Man (core M2), and GlcNAcβ1-4Man (core M3) (8) (Fig. 1*A*). Core M3 glycans are almost exclusively attached to alpha-dystroglycan (α-DG) (6, 7), and are extended by matriglycan. In contrast, Core M1 and M2 glycans are shorter *O*-Man glycans and are attached to many other glycoproteins. Core M1 is synthesized by POMGNT1, which transfers a GlcNAc residue to mannose *via* a β1-2 linkage (6, 7) (Fig. 1*A*). Core M2, the main focus in this study, is a brain-specific *O*-Man core structure carrying β1,6-GlcNAc branch (9) (Fig. 1*A*). This branch structure is synthesized by a brain-specific enzyme, *N*-acetylglucosaminyltransferase-IX (GnT-IX, also designated as MGAT5B) (10, 11) (Fig. 1*A*). GnT-IX was originally identified as a homolog of *N*-acetylglucosaminyltransferase-V (GnT-V, also designated as MGAT5), a GlcNAc transferase for *N*-glycan branching (10, 12) (Fig. 1*B*). The overall sequence identity between human GnT-IX and GnT-V is approximately 42%, with particularly high similarity within their catalytic domains (10). GnT-IX displays remarkably weaker activity toward *N*-glycans than GnT-V (10, 13) (Fig. 1*B*), while it shows much higher activity toward *O*-Man glycans than toward *N*-glycans (13). It was also reported that GnT-V-KO tissues and cells showed almost complete loss of the β1,6-GlcNAc branch in *N*-glycans (14). These findings indicate that GnT-IX and GnT-V primarily act on *O*-Man and *N*-glycans, respectively, *in vivo*. Nevertheless, the detailed mechanisms by which these highly similar enzymes differentially recognize their distinct acceptor substrates remain unclear.

**Figure 1:**
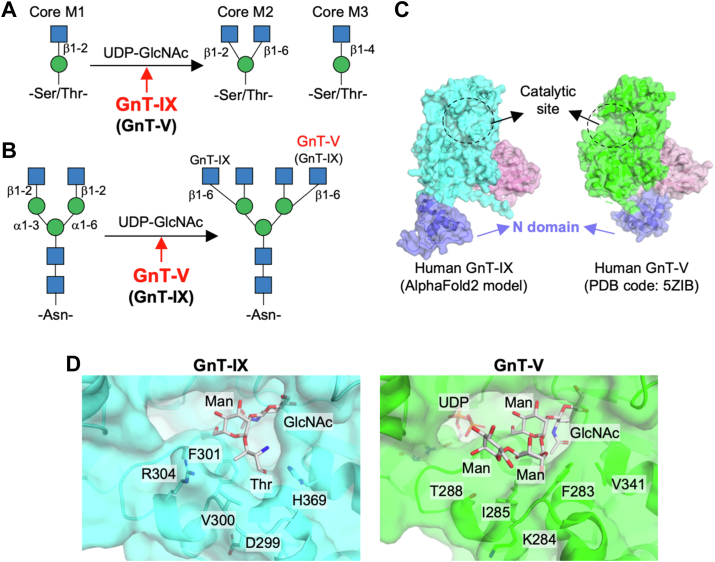
**Three-dimensional structures of the acceptor-substrate binding sites of GnT-IX and GnT-V**. *A*, schematic model of the GlcNAc transfer reaction toward *O*-Man glycan catalyzed by GnT-IX (MGAT5B) and three types of *O*-Man core structures. *B*, schematic model of the GlcNAc transfer reaction toward *N-*glycan catalyzed by GnT-IX and GnT-V (MGAT5B and MGAT5). *C*, the overall structure of GnT-IX (AlphaFold2) and GnT-V (PDB ID:5ZIB). *D*, the AlphaFold-predicted GnT-IX structure docked with a minimal *O*-Man acceptor, GlcNAcβ1-2Man-*O*-Thr (*left*) and the crystal structure of GnT-V in complex with an *N*-glycan acceptor pentasaccharide (GlcNAcβ1-2Manα1-6(GlcNAcβ1-2Manα1-3)Man) and UDP-GlcNAc (PDB: 6YJU) (*right*).

In addition to the branch formation, the mechanisms by which the subsequent elongation of *O*-Man glycans is regulated are poorly understood. Previous studies have shown that termini of brain *O*-Man glycans are modified with sialic acid, LewisX, human natural killer-1 (HNK-1), and keratan sulfate (KS) (9, 15, 16). Because these glycan epitopes are known to play key roles in high order brain functions, such as synaptic plasticity and axon regeneration (17, 18, 19), the biosynthesis of the *O*-Man glycan core could provide a platform for expressing various functional glycans in the brain. However, there have been limited studies on the enzymatic mechanisms of *O*-Man elongation *in vivo*. In particular, the effect of the branching of *O*-Man on subsequent elongation remains unexplored.

It was shown that the alteration in *O*-Man branching leads to the development or amelioration of brain disorders. GnT-IX-KO mice exhibited more rapid remyelination than wild-type (WT) mice after chemically induced demyelination (4). Furthermore, GnT-IX was reported to be involved in glioma, and knockdown of GnT-IX inhibits glioma growth *in vivo* (5). These pathological roles of branched *O*-Man glycans could be mediated by the terminal glycan epitopes in *O*-Man glycans; however, the precise mechanisms by which branched *O*-Man glycans regulate these phenomena remain unclear. Based on these findings, elucidation of the mechanism regulating the elongation of branched *O*-Man glycans contributes not only to unraveling the detailed mechanism by which *O*-Man glycans are synthesized but also to developing novel drugs against demyelination and brain tumors targeting *O*-Man glycans.

In this study, we explored the mechanisms of *O*-Man glycan recognition by GnT-IX and *O*-Man elongation enzymes. By structural comparison and mutagenesis, we identified the amino acid residue in GnT-IX that is critical to the specificity toward *O*-Man glycan acceptor. Using biochemical analysis of the GnT-IX-KO brain in combination with the *in vitro* enzyme assays, we further revealed that GnT-IX promotes KS biosynthesis on branched *O*-Man glycans. Our present findings deepen our understanding of the mechanism of maturation of branched *O*-Man glycans, highlighting the impact of branching on glycan biosynthesis.

## Results

### GnT-IX R304 is critical for the recognition of acceptor O-man glycan

To elucidate the mechanism that gives rise to the distinct acceptor substrate specificities of GnT-IX and -V, we first focused on the structures of the catalytic pocket of these enzymes with their respective acceptor substrates. In addition to the crystal structure of GnT-V in complex with an *N*-glycan acceptor pentasaccharide (GlcNAcβ1-2Manα1-6[GlcNAcβ1-2Manα1-3]Man) and UDP (PDB: 6YJU) (Fig. 1*D*, right), a GnT-IX-substrate complex model was created comprising the AlphaFold-predicted GnT-IX structure docked with a minimal *O*-Man acceptor, GlcNAcβ1-2Man-*O*-Thr (Fig. 1*D*, left). Based on these structures, we predicted the amino acid residues potentially involved in selective interaction with *N*- and *O*-Man glycans, respectively. In detail, D299-V300-F301, R304, and H369 in GnT-IX and F283-K284-I285, T288, and V341 in GnT-V possibly interact with the glycan core or aglycon of acceptors, contributing to the strict specificity toward the respective acceptor substrates. In particular, R304 in GnT-IX has a longer side chain than T288 in GnT-V, which could be suitable to accommodate the less bulky *O*-Man glycan core structure, and H369 is in a position where it could interact with a polypeptide moiety. In our previous study, we also experimentally showed that F283 and K284 in GnT-V are required for the activity toward *N*-glycan substrates (20). Based on these findings, we designed swap mutants in which these amino acids were interconverted between GnT-IX and GnT-V (D299-V300-F301 in GnT-IX vs. F283-K284-I285 in GnT-V, R304 in GnT-IX vs. T288 in GnT-V, and H369 in GnT-IX vs. V341 in GnT-V) and compared their activity toward *N*- and *O*-Man glycans.

**Figure 2:**
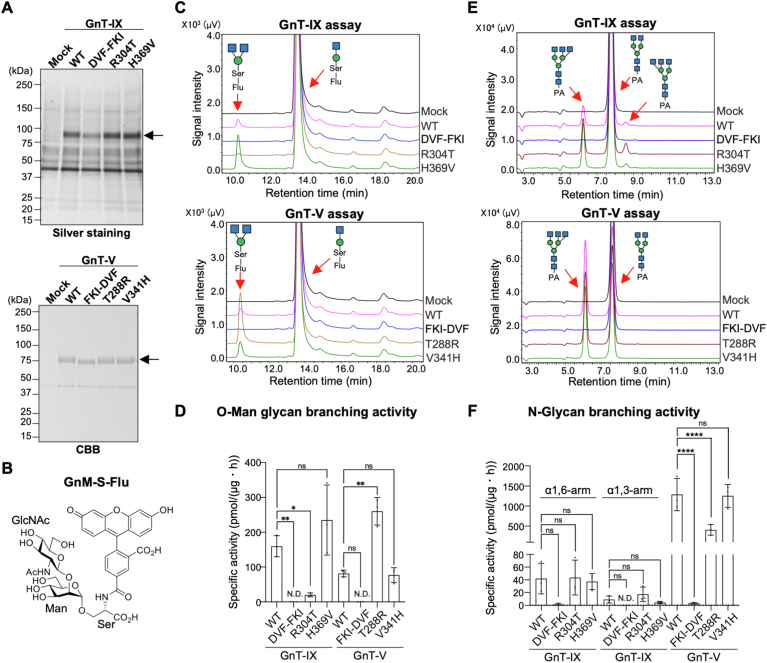
**Enzyme activity assays of mutants of GnT-IX and GnT-V**. *A*, soluble GnT-IX and -V, and their mutants were expressed in COS7 cells and purified from the medium using a Ni^2+^ column. Purified GnT-IX and -V enzymes were separated by SDS-PAGE and visualized by silver staining or CBB staining. *B*, The chemical structure of GnM-S-Flu that was synthesized and used for the *O*-Man-type acceptor substrate. *C*, purified GnT-IX, -V, and their mutants were incubated with GnM-S-Flu, and the reaction mixtures were analyzed by reverse-phase HPLC. *D*, the specific activities of GnT-IX, -V and their mutants toward GnM-S-Flu were calculated from the peak areas in (*C*) (n = 3, mean ± SD, ∗*p* < 0.05, ∗∗*p* < 0.01, Tukey’s multiple comparisons test). *E*, purified GnT-IX, -V, and their mutants were incubated with GnGnbi-PA, and the reaction mixtures were analyzed by reverse-phase HPLC. *F*, the specific activities of GnT-IX, -V and their mutants toward GnGnbi-PA were calculated from the peak areas in (*E*) (n = 3, mean ± SD, ∗∗∗∗*p* < 0.0001, Tukey’s multiple comparisons test).

His-tagged soluble truncated forms of the WT and mutant enzymes were expressed in COS7 cells and purified using a Ni^2+^-column (Figs. 2*A* and S1), and these purified enzymes were used for *in vitro* enzyme assays. As an *N*-glycan acceptor, the pyridylamine (PA)-labeled GlcNAc-terminated biantennary *N*-glycan (GnGnbi-PA) was used in accordance with a previously established method (21). As an *O*-Man glycan acceptor, we chemically synthesized fluorescein-labeled GlcNAcβ1-2Man-Ser (GnM-S-Flu) (Fig. 2*B*, S2 and S3) based on the previously reported method (22). These fluorescent substrates were incubated with the purified enzymes, and the reaction mixtures were analyzed using HPLC to separate the substrates and products and to determine the enzyme activity.

First, we measured the enzyme activity toward GnM-S-Flu (Fig. 2, *C* and *D*). Upon incubation with GnT-IX WT, we detected the product peak, and we confirmed by MALDI-TOF-MS that it had an additional single GlcNAc residue compared with the substrate as expected (Fig. S4, G4), demonstrating that the synthesized substrate can be used to measure *O*-Man branching activity. In addition, we observed that GnT-V WT gave a product peak with the same retention time as that of GnT-IX, and the specific activity of GnT-V toward the *O*-Man substrate was approximately half that of GnT-IX (Fig. 2*D*). This is consistent with a previous report showing that GnT-V has weak but detectable activity toward *O*-Man substrate as well as toward *N*-glycan substrate and partially compensates for the loss of GnT-IX activity in GnT-IX-KO mice (23). For the mutants, GnT-IX DVF-FKI and GnT-V FKI-DVF completely lost their activity, indicating the importance of these residues for substrate recognition. Considering the possibility that the conversion of 3 amino acids in GnT-IX DVF-FKI and GnT-V FKI-DVF caused an unexpected global structural defect, we further created GnT-IX D299 F and GnT-V F283D mutants (Fig. S5*A*). We observed significant decreases in the activity for both mutants (Fig. S5, *B* and *C*), showing that these residues are crucial for GnT-IX and GnT-V. Remarkably, R304T mutation in GnT-IX showed a dramatic reduction in activity, while GnT-V T288R conversely showed a 4-fold increase in activity compared with WT. These results suggest that R304 of GnT-IX is critical for determining the preference for *O*-Man glycan substrate.

Next, we analyzed the enzyme activity toward GnGnbi-PA (Fig. 2, *E* and *F*). Consistent with previous reports, GnT-IX gave peaks for two products, one with a β1,6-branch on the α1,6-Man arm, the same as GnT-V, and the other with a β1,6-branch on the α1,3-Man arm, a GnT-IX-specific product (10). In addition, the specific activity of GnT-IX for both products was found to be markedly lower than that of GnT-V (Fig. 2*F*). For the mutants, DVF-FKI, FKI-DVF, D299F, and F283D again abolished the activity (Figs. 2*F* and S5*C*). The other mutants did not show pronounced changes in the specific activity or branch specificity toward *N*-glycan substrate, suggesting that amino acids other than the swapped residues could also be involved in recognition of the *N*-glycan substrate and that the preference for the *N*-glycan substrate cannot be switched by only swapping these residues between GnT-IX and V.

**Figure 3:**
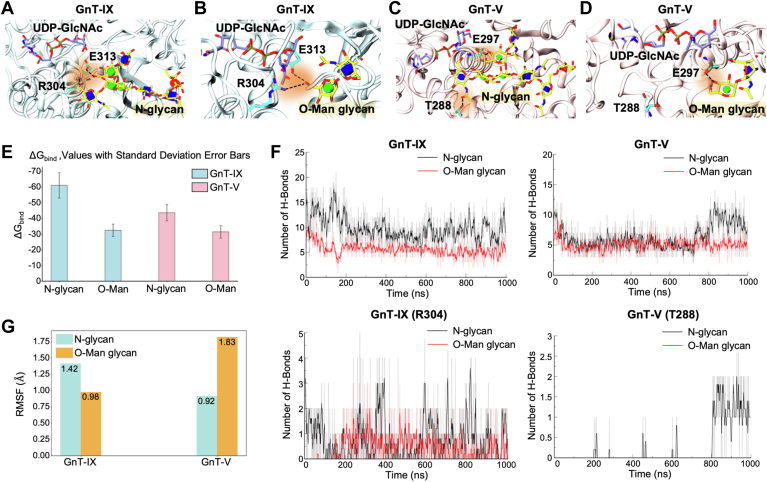
**Binding modes of *N*- and *O*-Man glycan acceptors in GnT-IX and GnT-V**. *A–D*, proteins are colored in *blue* (GnT-IX) or *pink* (GnT-V), and glycans are colored in *yellow*. The interactions are shown in *black* and highlighted in *orange*. The complex structures of GnT-IX with *N*-glycan (*A*) or with *O*-Man glycan (*B*), and GnT-V with *N*-glycan (*C*) or with *O*-Man glycan (*D*) are shown. *E*, the MM/GBSA energies associated with the binding of acceptor glycans to GnT-IX or GnT-V as calculated using the GB1 model of MM/GBSA in Amber24. *F*, the total number of hydrogen bonds formed by the glycans with GnT-IX and -V (*upper*) or with their specified residue(s) (*lower*). *G*, RMSF of the acceptor Man residue in *N*- and *O*-Man glycan substrates in complex with GnT-IX or GnT-V.

To corroborate the enzyme assay results and study the roles of R304 (GnT-IX) and T288 (GnT-V) in catalytic activity, we next performed molecular dynamics (MD) simulations and binding energy calculations for acceptor glycans (*N*- and *O*-Man glycan) bound to GnT-IX and GnT-V (Fig. 3, *A*–*D*). The MD simulations and MM/GBSA binding energy analyses showed that the acceptor *N*-glycan binds to GnT-V with a comparatively stronger binding energy (−43.7 ± 5.1 kcal mol^-1^) than to *O*-Man (−31.4 ± 4.0 kcal mol^-1^) (Fig. 3*E*). This difference in binding energy suggests that *N*-glycan can effectively position the acceptor mannose residue in the catalytic site of GnT-V. A notable difference was seen in the spatial arrangement of T288, which interacts and forms a hydrogen bond with the GlcNAc moiety of the α1,3-branch of the *N*-glycan (Figs. 3*C* and S6*A*). In contrast, T288 of GnT-V does not directly interact with *O*-Man (Figs. 3*D* and S6*B*).

In GnT-IX, R304 occupies the same position as T288 but forms significantly more favorable electrostatic interactions with *N*-glycan than T288 in GnT-V does (Figs. 3*A* and S6*C*). As a result of these changes in molecular interactions, *N*-glycan acceptors achieve a much stronger binding energy in GnT-IX (−61.1 ± 8.1 kcal mol^-1^) than in GnT-V (−43.7 ± 5.1 kcal mol^-1^) (Fig. 3, *E* and *F*). Although stronger binding is often assumed to yield better kinetics, our data indicate the opposite for GnT-IX with *N*-glycans. There are two plausible reasons for this: (1) excessive stabilization of the *N*-glycan results in restricted conformational mobility near the catalytic site, which may prevent the reorientation required for in-line nucleophilic attack, thereby lowering the activity; and (2) affinity beyond an optimal threshold prolongs product residence time, slowing the turnover.

The *O*-Man binding in GnT-IX (−32.4 ± 4 kcal mol^-1^) is marginally stronger than in GnT-V (−31.4 ± 4.0 kcal mol^-1^) (Fig. 3*E*), aligning with their relative activity in the enzyme assays. The R304 forms a hydrogen bond with O6 of Man in *O*-Man and contributes to acceptor binding (Fig. 3, *B* and *F*, S6*D*, and Table S1). We further calculated the root mean square fluctuations (RMSF) of the acceptor Man in both *N*-glycan and *O*-Man, and it is evident that lower fluctuations of the Man residue at the glycosylation site correlate with higher enzymatic catalytic activity. Specifically, GnT-IX stabilized Man of *O*-Man glycan (RMSF ≈ 0.98 Å), while GnT-V stabilized Man of *N*-glycans (RMSF ≈ 0.92 Å), with much lower RMSF values than those of the *N*-glycan in GnT-IX (1.42) and *O*-Man in GnT-V (1.83) (Fig. 3*G*). RMSF of the acceptor Man suggests that stabilization of the acceptor Man in the catalytic pocket is a key determinant of efficient GlcNAc transfer to the preferred substrates (*O*-Man glycan for GnT-IX and *N*-glycan for GnT-V).

### KS is attached to branched O-Man glycans on phosphacan in the brain

**Figure 4:**
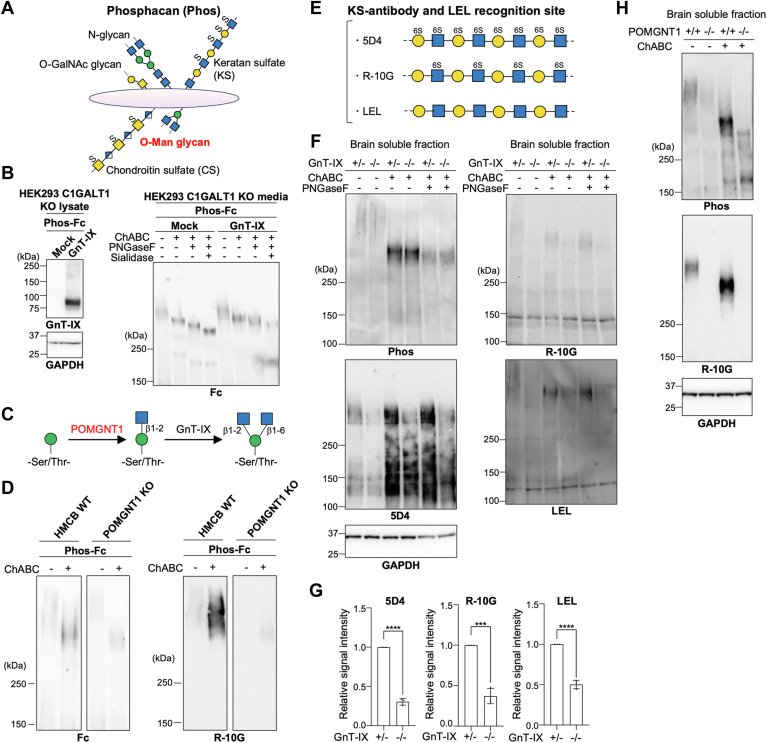
**The levels of KS in GnT-IX-KO and POMGNT1-KO mouse brains**. *A*, schematic diagram of phosphacan. *O*-Man glycans, *N*-glycans, *O*-GalNAc glycans (mucin-type glycans), chondroitin sulfate (CS), and keratan sulfate (KS) are present on phosphacan. *B*, Phosphacan-Fc (Phos-Fc) and GnT-IX were expressed in HEK293 C1GALT1 KO cells. (*Left*) Cell lysates were western blotted for GnT-IX and GAPDH. (*Right*) Phosphacan was purified from the media using Dynabeads protein G. After treatment of phosphacan with ChABC, PNGaseF, and sialidase, proteins were separated by SDS-PAGE and blotted with anti-human IgG. *C*, schematic model of the reactions catalyzed by POMGNT1 and GnT-IX. *D*, Phosphacan was expressed in HMCB WT and POMGNT1-KO cells (clone #3 in Fig. S8) and purified from the media. After treatment of phosphacan with ChABC, proteins were separated by SDS-PAGE and blotted with anti-human IgG and R-10G. *E*, representative glycan structures recognized by 5D4 and R-10G antibodies and LEL lectin. *F*, blotting of proteins in the soluble fractions from GnT-IX (*Mgat5b*) heterozygous and KO mouse brains (postnatal day 0 [P0]) with anti-phosphacan, 5D4, R-10G, anti-GAPDH or LEL lectin. *G*, signal intensities of the band corresponding to the position of phosphacan in 5D4, R-10G, and LEL blots in (*F*) were quantified, and adjusted for GAPDH. The relative levels are shown as a graph (n = 3, mean ± SD, ∗∗∗*p* < 0.001, ∗∗∗∗*p* < 0.0001, unpaired *t* test). *H*, blotting of proteins in the soluble fractions from WT and POMGNT1-KO mouse brains (female, 4-week-old) with anti-phosphacan, R-10G, or anti-GAPDH.

Next, we investigated the elongation of *O*-Man glycan in the brain, where GnT-IX is almost exclusively expressed (24). It has previously been reported that termini of brain *O*-Man glycans are modified with Sia, LewisX, HNK-1, and KS (9, 15, 16), most of which are known to be attached to the chondroitin sulfate proteoglycan phosphacan (Fig. 4*A*), or its receptor-type isoform PTPRZ (9). Phosphacan/PTPRZ is heavily glycosylated with *O*-Man glycans (5), *N*-glycans (19 sites), *O*-GalNAc glycans (9), chondroitin sulfate (CS) (5 sites, Uniprot: P23471), and keratan sulfate (KS). Although *O*-linked HNK-1 was reported to be reduced in GnT-IX-KO mouse brain to about half the level of WT (4), little is known about whether the amounts of other terminal epitopes in *O*-Man glycans depend on branching.

To characterize the elongation of *O*-Man glycans on phosphacan, we first expressed Fc-tagged phosphacan (phosphacan-Fc) in HEK293 C1GALT1-KO cells (25), which lack mucin-type glycans except the reducing end GalNAc residue. Expressed phosphacan-Fc was purified from the culture media and detected by western blotting after treatments with chondroitinase ABC (ChABC), PNGaseF, and Sialidase (Fig. 4*B*, right). The successive downshift of phosphacan in SDS-PAGE gel upon glycosidase treatment and the loss of signal with *N*-glycan-reactive leukoagglutinating-phytohemagglutinin (L4-PHA) (26) after PNGaseF treatment (Fig. S7*A*) suggest that expressed phosphacan is sialylated on its glycans other than *N*- and mucin-type *O*-glycans. Furthermore, when GnT-IX was co-expressed, the phosphacan band was shifted upward compared with that in the mock control, even after PNGaseF and sialidase treatments (Fig. 4*B* right, fourth and eighth lanes). This suggests that phosphacan is *O*-mannosylated in cells and that its *O*-Man glycans are modified by GnT-IX.

We further characterized *O*-Man glycans on phosphacan using another cell line, human melanoma HMCB, which endogenously expresses neural glycan epitope HNK-1 (27). We also established HMCB KO cells lacking POMGNT1 (Fig. S8*A*), which is required for *O*-Man glycan biosynthesis (Fig. 4*C*). The abolition of *O*-Man elongation in POMGNT1-KO cells was confirmed by loss of laminin-reactive matriglycan on α-DG (8) in these cells (Fig. S8*B*). When expressed in HMCB WT cells, purified phosphacan-Fc showed the broad band pattern even after ChABC treatment (Fig. 4*D* left, second lane), and blocking *O*-Man elongation by deleting POMGNT1 resulted in a large downshift (Fig. 4*D* left, second and fourth lanes, and S8C). Furthermore, when probed with R-10G, an antibody against KS (Fig. 4*E*), the signal on phosphacan was decreased in POMGNT1-KO cells (Fig. 4*D*, right). We further confirmed that the signal with R-10G was not detected in phosphacan-Fc (−) control (Fig. S7*B*) and that the signal with R-10G on phosphacan was resistant to PNGaseF but sensitive to a polylactosamine-digesting enzyme endo-β-galactosidase (Fig. S7*B*, sixth lane). These findings indicate that KS is attached to *O*-Man glycans on phosphacan, and we further focused on the impact of *O*-Man branching by GnT-IX on the level of KS.

KS is a sulfated polysaccharide with a polylactosamine backbone, and KS and polylactosamine can be detected with 5D4 mAb, R-10G mAb, and Lycopersicon Esculentum lectin (LEL) (Fig. 4*E*) (28, 29, 30). Because KS is abundantly expressed in embryonic and juvenile brain (31), we analyzed brain-soluble fractions from GnT-IX-KO and control GnT-IX heterozygous mice at postnatal day 0 using antibodies against phosphacan and KS, and LEL. The results showed that, after ChABC treatment, the signals with 5D4, R-10G, and LEL were significantly reduced in GnT-IX-KO mice (Fig. 4, *F*and *G*), particularly the 5D4 and R-10G signals being approximately one-third of those in control GnT-IX heterozygous mice. Moreover, digestion with PNGaseF, which cleaves *N*-glycans, did not reduce the signals (Fig. 4*F*), and we also found that the R-10G signal at the position corresponding to phosphacan was lost in POMGNT1-KO mouse brain (Fig. 4*H*). These results demonstrated that the level of KS attached to phosphacan in the mouse brain largely depends on the branching of *O*-Man glycans.

### Enzymes for KS biosynthesis prefer branched O-Man glycans as substrates

**Figure 5:**
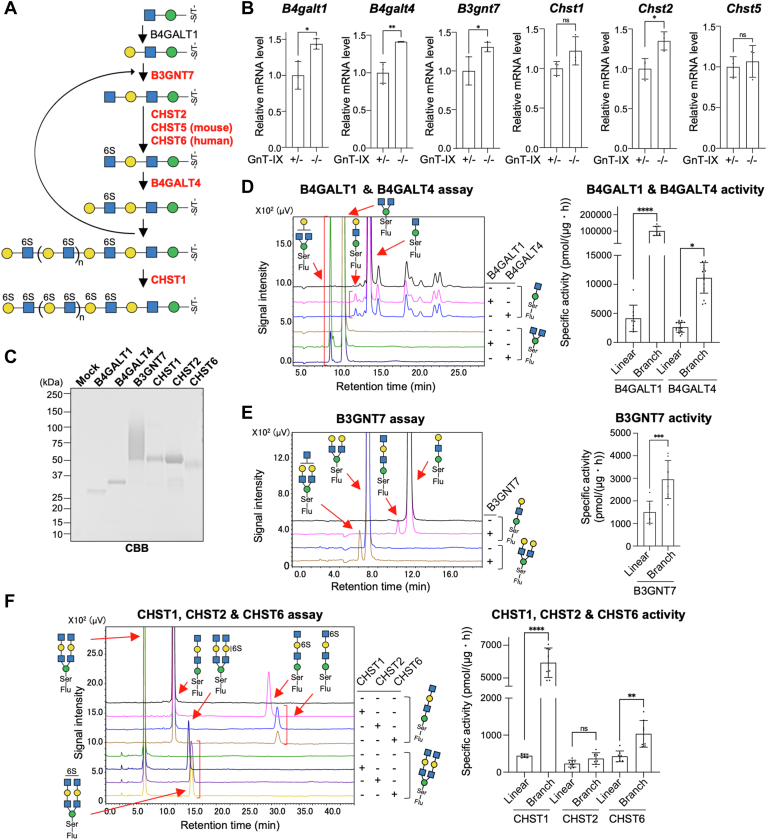
**Enzyme assays of KS biosynthetic enzymes toward linear and branched *O*-Man glycans**. *A*, enzymes involved in the KS biosynthesis. *B*, qPCR analysis of *B4galt1*, *B4galt4*, *B3gnt7*, *Chst1*, *Chst2* and *Chst5* mRNAs in GnT-IX-KO mouse brains (P0) was performed, and their relative levels normalized by that of *Gapdh* are shown as a graph (n = 3, mean ± SD, ∗*p* < 0.05, ∗∗*p* < 0.01, unpaired *t* test). *C*, soluble B4GALT1, B4GALT4, B3GNT7, CHST1, CHST2, and CHST6 were expressed in HEK293 T cells and purified from the media using a Ni2+ column. The purified enzymes were separated by SDS-PAGE and visualized by CBB staining. *D*–*F*, Purified B4GALT1, B4GALT4, B3GNT7, CHST1, CHST2, and CHST6 were incubated with GnM-S-Flu or Gn(Gn)M-S-Flu (for B4GALT1 and B4GALT4) (*D*), GalGnM-S-Flu or GalGn(GalGn)M-S-Flu (for B3GNT7) (*E*), or GnGalGnM-S-Flu or GnGalGn(GnGalGn)M-S-Flu (for CHSTs) (*F*), and the reaction mixtures were analyzed by reverse-phase HPLC. The amount of CHST1 used for GnGalGn(GnGalGn)M-S-Flu was one-20th of the amount for GnGalGnM-S-Flu, while the same amounts of the other enzymes were used for linear and branched substrates. The positions of sulfate residue were predicted by the substrate specificity of the enzymes. The specific activities were calculated from the peak areas (n = 8–17, mean ± SD, ∗*p* < 0.05, ∗∗*p* < 0.01, ∗∗∗*p* < 0.001, ∗∗∗∗*p* < 0.0001, Holm-Sidak’s multiple comparison test for B4GALT1, B4GALT4, CHST1, CHST2, and CHST6, and unpaired *t* test for B3GNT7).

Based on the substantial reduction in the level of KS in GnT-IX-KO mice, we hypothesized that the KS biosynthesis is diminished by the loss of *O*-Man branching. KS is sequentially biosynthesized by B3GNT7, CHST2 and CHST6, B4GALT1 and B4GALT4, and CHST1 (15) (Fig. 5*A*). Notably, human CHST6 corresponds to mouse CHST5, and knocking out CHST5 in mice resulted in almost complete disappearance of the R-10G signal on phosphacan (29), showing that CHST5 is a crucial enzyme for KS biosynthesis in mouse brain. To first test the possibility that either one of these KS biosynthetic enzymes is abnormally downregulated in GnT-IX-KO mice, we compared the expression levels of *B4galt1*, *B4galt4*, *B3gnt7*, *Chst1*, *Chst2*, and *Chst5* mRNAs in the mouse brain. qPCR analysis confirmed that the expression levels of genes encoding these glycosyltransferases and sulfotransferases were not decreased in GnT-IX-KO mice (Fig. 5*B*), ruling out the possibility that the KS reduction in GnT-IX-KO mice is due to impaired expression of the genes encoding biosynthetic enzymes. We then reasoned that either one of these enzymes preferentially acts on the branched *O*-Man glycans compared with the linear *O*-Man glycans.

To test this idea, we first expressed and purified all of these KS biosynthetic enzymes from HEK293T culture media as soluble forms for the *in vitro* activity assays (Fig. 5*C*). The broad band pattern of B3GNT7 is due to self-modification with polylactosamine, as the bands converged upon treatment with endo-β-galactosidase (Fig. S9). Next, we prepared both linear and branched *O*-Man acceptor substrates for all of these enzymes by enzymatic synthesis using GnM-S-Flu as the starting material (Fig. S4*A*), and all of the purified substrates were confirmed by MALDI-MS to have the expected masses (Fig. S4*B*). In the activity assays, to compare the initial reaction rate, we measured the activity with the controlled reaction times and enzyme amounts for the transfer of a single sugar or a sulfate residue, even toward the branched acceptor substrates.

Figure 5, *D*–*F* shows the results of the activity assays. Remarkably, B4GALT1 and B4GALT4 showed higher activity toward the branched substrate than toward the linear substrate, with 23.6-fold and 4.49-fold increases, respectively (Fig. 5*D*). This is well consistent with our MD simulations showing that the branched *O*-Man acceptor binding to B4GALT1 is stronger (−27.0 ± 5.7) than that for the linear *O*-Man (−9.9 ± 8.2) *via* strong electrostatic interaction with R355 of B4GALT1 (Fig. S10). We further performed kinetic analysis and found the significantly lower *K*_m_ and higher *k*_cat_ values of B4GALT1 for the branched substrate than the linear counterpart (Fig. S11). In contrast, the activity of B3GNT7, CHST2, and CHST6 was approximately twice as high toward the branched substrates as toward the linear ones (Fig. 5, *E* and *F*, S12), but this could simply have been due to the presence of twice the number of acceptor sites in the branched substrates than in their linear counterparts. Remarkably, CHST1 showed 13.4-fold higher activity toward the branched substrate than toward the linear one (Figs. 5*F* and S12). These findings revealed that B4GALT1, B4GALT4, and CHST1 have stronger preferences for branched *O*-Man glycans than for their linear counterparts. This could likely explain why the KS levels are reduced in GnT-IX-KO mouse brain, suggesting the role of the branch of *O*-Man glycans as an efficient scaffold for glycan elongation to produce highly sulfated KS in the brain.

## Discussion

**Figure 6:**
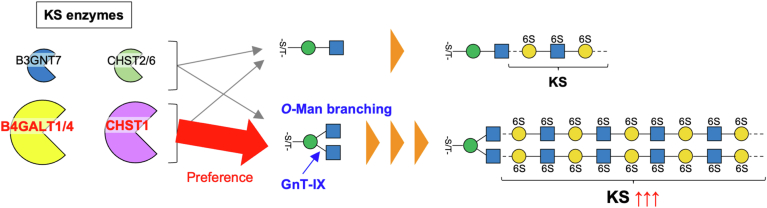
**Actions of KS biosynthetic enzymes on *O*-Man glycans**. B4GALT1, B4GALT4, and CHST1 preferentially act on GnT-IX-produced branched *O*-Man glycans.

Our enzyme assays of GnT-IX and -V in combination with structure-guided mutation showed the importance of R304 in GnT-IX for determining the preference of GnT-IX for *O*-Man glycans (Fig. 2, *C* and *D*). In addition, we demonstrated that the specific enzymes involved in KS biosynthesis, B4GALT1, B4GALT4, and CHST1, react with the GnT-IX-produced branched *O*-Man glycans remarkably more rapidly than with the linear *O*-Man (Fig. 6), which is consistent with the significant decrease in the KS level in GnT-IX-KO brain (Fig. 4*G*). Our intensive biochemical assays using the purified enzymes and various types of *O*-Man substrates provided evidence for the role of *O*-Man branching for KS extension in the brain by serving as the preferred scaffold for biosynthesis. Together with the previous finding that the level of matriglycan on α-DG is unchanged in GnT-IX-KO mice (23), our data suggest that GnT-IX regulates the extension of core M2-type *O*-Man glycans.

Notably, B4GALT1 strongly favors branched *O*-Man glycans rather than the linear *O*-Man (Figs. 5*D*, S10 and S11). Considering that B4GALT1 is broadly involved in the biosynthesis of many terminal glycan epitopes, this raises the possibility that the other terminal epitopes, such as HNK-1, polylactosamine, and LewisX on *O*-Man glycans could also be enhanced by the action of GnT-IX. A previous study reported that *O*-linked HNK-1 glycan in GnT-IX-KO mouse brains decreased to approximately half of the WT level (4), and we also showed that the level of polylactosamine decreases to about half that of heterozygous mice (Fig. 4*G*). This suggests that unlike the above KS enzymes (B4GALT1, B4GALT4, and CHST1), the biosynthetic enzymes for HNK-1 and polylactosamine, such as GlcAT-P (B3GAT1) and HNK-1ST (CHST10) (32), and B3GNT2 and B3GNT8 (33), respectively, do not have such a strong preference for the branched substrate. To examine the more general role of *O*-Man branching for its extension, it is necessary to investigate the detailed substrate specificity of many biosynthetic enzymes for terminal structures and to analyze the *in vivo* changes in other glycans in the brains of GnT-IX-KO mice.

Regarding the substrate specificity of KS enzymes, we here used the short KS components for enzyme assays (Fig. 5). However, because KS exists in a polymerized state in the brain, the biosynthetic enzymes might have different activity toward longer KS chains *in vivo*. Indeed, during enzyme preparation, we noticed that no activity of CHST1 was detected toward the shorter GalGnM-S-Flu. In future work, it would be intriguing to prepare longer linear and branched KS substrates to examine more *in vivo*-like substrate preferences (linear vs. branched) of the KS enzymes. We also found that some of the KS enzymes, when reacted with the branched substrates, unevenly gave the two products that have the product monosaccharide or sulfate on different arms (Fig. S13). For instance, B4GALT1 and B4GALT4 gave the products with a larger peak at a shorter retention time (Fig. S13*A*). CHST1 and CHST6 appeared to give only a single product peak, whereas B3GNT7 and CHST2 gave the two products at comparable levels (Fig. S13, *B*–*D*). This suggests that B4GALT1, B4GALT4, CHST1, and CHST6 preferentially act on either the β1-2 arm or the β1-6 arm. To further understand how branched KS chains are sequentially biosynthesized in the brain, future work will be needed to analyze the detailed structure of the dominant product of each KS enzyme, to elucidate its arm preference, and to establish the method to analyze the detailed structures of brain-derived KS from WT and GnT-IX-KO mice.

In addition to the oligosaccharide structures, the polypeptide moiety of the substrate likely influences the biosynthesis of branched *O*-Man glycans. A previous biochemical study showed that GnT-IX prefers an *O*-Man glycopeptide rather than benzyl-Man-GlcNAc in *in vitro* enzyme assays (13). Furthermore, we reported that the luminal non-catalytic N-terminal domain (N-domain) of GnT-V is crucial for recognizing the polypeptide moiety of substrate glycoproteins (34). GnT-IX also possesses an N-domain, but its structure differs from that of GnT-V (Fig. 1*C*, purple). Notably, we recently discovered that GnT-V selectively modifies two major glycoproteins in the mouse kidney (35), indicating that GnT-V has strict substrate protein selectivity *in vivo*. These findings suggest that GnT-IX also recognizes the polypeptide part of the substrate glycoproteins to enable protein-selective modification. Because only a few glycoproteins (PTPRZ/Phosphacan, Neurofascin186, and CD24) have been identified to be modified with branched *O*-Man glycans (4, 36, 37) owing to the lack of a specific probe, it is important to develop a new technique to identify the substrate proteins of GnT-IX. Such analysis could deepen our understanding of the mechanism of GnT-IX catalysis *in vivo*.

A previous study reported that GnT-IX inhibits remyelination (4), and it was proposed that modification of PTPRZ (a receptor-type isoform of phosphacan) by GnT-IX and subsequent regulation of the surface localization of PTPRZ are involved in this process. Another study reported that the removal of chondroitin sulfate on PTPRZ resulted in its punctate localization on the cell surface, leading to its inactivation and oligodendrocyte differentiation (38). This suggested that a large negatively charged glycosaminoglycan on PTPRZ impacts its functions (38). Our data implied a significant reduction in KS on phosphacan in GnT-IX-KO mice, raising a possibility that a similar KS reduction occurs on membrane-bound PTPRZ. The decrease in the KS level might cause an effect similar to that of chondroitin sulfate, that is, clustering of PTPRZ on the cell surface thereby suppressing its activity and enhanced remyelination. Previous studies also revealed that KS inhibits axonal regeneration after nerve injury (18) and that KS is involved in neurodegenerative diseases such as Alzheimer's disease and amyotrophic lateral sclerosis (28, 39). Therefore, enhanced KS extension by *O*-Man branching could also play vital roles in these processes and diseases. In future work, it would be intriguing to examine the phenotypes of GnT-IX-KO mice in the context of axonal injury and neurodegeneration, which could lead to the development of a new drug target for these diseases.

In conclusion, our study elucidated at least some of the enzymatic basis for the branching and extension of *O*-Man glycans in the brain. Considering that GnT-IX and KS are implicated in various brain diseases, elucidating the detailed mechanisms by which GnT-IX recognizes *O*-Man glycans and by which *O*-Man glycans are further extended in the brain could provide new clues for the development of new diagnostic and therapeutic approaches targeting GnT-IX.

## Experimental procedures

### Reagents

The following antibodies and lectins were used: mouse anti-GAPDH (Merck Millipore; MAB374), rabbit anti-MGAT5B (Novus Biologicals; NBP3-05134), rabbit anti-Phosphacan (abcam; EPR26271–75), 5D4 (Merck; MABN2483), R-10G (TCI Chemicals; A2968), anti-Laminin (Sigma; L9393), HRP-anti-mouse IgG (GE Healthcare; NA931 V), HRP-anti-human IgG (anti-Fc tag) (Southern Biotechnology Associates; 6145–05), HRP-anti-rabbit IgG (GE Healthcare; NA934 V), biotinylated LEL (Vector Laboratories; B-1175–1), and L4-PHA (J-Chemical; J112). L4-PHA was labeled with HRP using a Peroxidase Labeling Kit-NH2 (Dojindo; LK11).

### Plasmid construction

Oligonucleotides for plasmid construction are listed in Table S2. pcDNA6.2DEST/human GnT-IX-FLAG (40) was constructed previously and used for transfection and PCR amplification as a template. For expressing soluble truncated glycosyltransferases and sulfotransferases, pcDNA-IH (41) and pcDNA-IHT were used. pcDNA-IHT was constructed by inserting an oligonucleotide encoding a TEV cleavage site into the EcoRI site of pcDNA-IH by Gibson assembly. For constructing pcDNA-IHT/human GnT-IX (R153 to C-term), a part of the GnT-IX cDNA was amplified by PCR and inserted into the EcoRI-EcoRV sites of pcDNA-IHT by Gibson assembly. Point mutations (D299 F, DVF-FKI, R304 T, and H369 V for GnT-IX, and F283D, FKI-DVF, T288 R, and V341H for GnT-V) were introduced by PCR using pcDNA-IHT/human GnT-IX and pcDNA-IH/human GnT-V (T121 to C-term) (42) as templates using the QuikChange Lightning Site-Directed Mutagenesis Kit.

cDNA for full-length human B4GALT1 was amplified by PCR using reverse-transcribed total RNA from HEK293 cells as a template and cloned into pCR-BluntII-TOPO by using Zero Blunt TOPO PCR Cloning Kit (Invitrogen). cDNAs for full-length human B4GALT4, B3GNT7, CHST1, CHST2, and CHST6 were amplified by PCR using reverse-transcribed total RNA from HMCB cells (for B4GALT4, B3GNT7, and CHST6) or LN-229 cells (for CHST2), or a human brain cDNA library (TAKARA, human MTC panel 1) (for CHST1) and cloned into the EcoRI site of pcDNA6-mycHisA by Gibson assembly to construct pcDNA6-mycHisA/B4GALT4, B3GNT7, CHST1, CHST2 and CHST6. To construct the plasmids of pcDNA-IHT/human B4GALT1 (L127 to C-term), pcDNA-IHT/human B4GALT4 (L74 to C-term), pcDNA-IH/human B3GNT7 (L35 to C-term), pcDNA-IH/human CHST1 (G41 to C-term), pcDNA-IH/human CHST2 (A118 to C-term), and pcDNA-IH/human CHST6 (A35 to C-term), fragments amplified by PCR using pCR-BluntII-TOPO-B4GALT1, pcDNA6-mycHisA/B4GALT4, B3GNT7, CHST1, CHST2, and CHST6 as templates were inserted into EcoRI/XhoI sites of pcDNA-IHT for B4GALT1 and B4GALT4, and the EcoRI site of pcDNA-IH for B3GNT7, CHST1, CHST2, and CHST6 by Gibson assembly.

p3xFLAG-CMV14/Phosphacan-myc-Fc was constructed by simultaneously inserting the longer fragment encoding Phosphacan-myc digested from pcDNA3.1/Phosphacan-myc-His (43) with EcoRV and AgeI and the shorter fragment encoding IgG2-Fc digested from pFUSE-hIgG-Fc1 with AgeI and NheI into EcoRV/XbaI sites of p3xFLAG-CMV14. The plasmid for mouse α-DG-Fc was kindly provided by Dr Kazuhiro Kobayashi (Kobe University) (44). To construct the plasmids for the generation of KO cells, oligonucleotide pairs were inserted into the BbsI site of the p x 330-puro vector.

### Cell culture

HEK293 (ATCC), HEK293 T (ATCC), COS7 (RIKEN Cell Bank), HEK293 C1GALT1-KO, HMCB (ATCC), HMCB POMGNT1-KO, and LN-229 (ATCC) cells were grown at 37 °C under 5% CO_2_ conditions in DMEM supplemented with 10% fetal bovine serum and 50 μg/ml kanamycin. HEK293 C1GALT1 KO cells were established as previously described (25).

### Generation of POMGNT1-KO cell lines

HMCB cells were transfected with two px330-puro plasmids encoding two distinct sgRNAs targeting human *POMGNT1*. The next day, cells expressing sgRNAs and Cas9 were selected upon incubation with 4 μg/ml puromycin for 1 day. After this selection, surviving cell clones were isolated by limiting dilution, and the genotypes of clones were validated by PCR with the following primer set: AAGAATACAGGAGCACTGGG and GCCATCCACTGCCACATATA. HMCB POMGNT1-KO cell clones #1 and #2 were generated using px330-puro_POMGNT1#1 and px330-puro_POMGNT1#2, and another KO cell clone #3 was generated using px330-puro_POMGNT1#2 and px330-puro_POMGNT1#3 (Table S2).

### Plasmid transfection

Cells cultured on a 10-cm or 6-cm dish were transfected with 10 μg or 5 μg of plasmid using Lipofectamine 3000 Transfection Reagent (Thermo Fisher Scientific), in accordance with the manufacturer’s protocol. For the expression of recombinant soluble GnT-IX, GnT-V, B4GALT1, B4GALT4, B3GNT7, CHST1, CHST2, and CHST6, 15 μg of the plasmids was transfected into COS7 or HEK293T cells cultured on a 15-cm dish using Polyethyleneimine MAX (Polyscience).

### Structural representation

The atomic structure of human GnT-IX was generated by AlphaFold2 (45). The docking model of *O*-mannosylated peptide in human GnT-IX was built based on the superposition of human GnT-V-UDP-acceptor complex (PDB ID: 6YJU, (46)). The coordinates of *O*-mannosylated peptide were modified with POMGNT2-*O*-mannosylated peptide complex (PDB ID: 7E9L, (47)). Structural superposition was performed with the program SUPERPOSE (48). The structural figures were drawn with PyMOL (The PyMOL Molecular Graphics System, Version 3.1.4.1 Schrödinger, LLC.).

### Thermostability assay

The thermal shift assay was performed using Tycho NT. 6 (NanoTemper Technologies). Purified GnT-IX WT and the mutants (DVF-FKI, R304 T, and H369 V), and GnT-V WT and the mutants (FKI-DVF, T288 R, and V341H) in 50 mM MES buffer (pH 6.25) were loaded in glass capillaries. Intrinsic fluorescence was recorded at 330 nm and 350 nm while heating the sample from 35 − 95 °C. The ratio of fluorescence (350/330 nm) and the inflection temperatures (Ti) were calculated by Tycho NT.6.

### MD simulation

The structures of the catalytic domain models of human GnT-V (MGAT5; PDB ID 6YJU (46)) and B4GALT1 (PDB ID: 4EEO (49)) were obtained from protein data bank. The structure of GnT-IX (MGAT5B) was obtained from the AlphaFold database (50). The structures of *N*-glycan (GlyTouCan ID: G80858 MF), linear *O*-Man (GlyTouCan ID: G85856 KC), and branched *O*-Man (GlyTouCan ID: G56868BH) were prepared using Glycam-Web (51). The structure of UDP-GlcNAc was obtained from the GnT-V crystal structure and included in all GnT-IX and GnT-V simulations. The structure of UDP-Gal was obtained from PDB ID: 4EEO (49) and included in the B4GALT1 simulations. Enzyme complexes with acceptor glycans were assembled by aligning the reactive Man with the Man of the pentasaccharide in the GnT-V crystal structure. The systems were solvated in OPC water in octahedral boxes with 10 Å padding and neutralized with NaCl. Simulations were performed in Amber24 using Amber ff19SB (52) for proteins, GLYCAM-06j-1 (revision j-1) (53) for glycans and GAFF2 (54) with RESP charges (HF/6–31G∗) for UDP-GlcNAc.

The systems were equilibrated using a previously published multistep protocol (55). All systems were heated from 0 to 300 K using a Langevin Thermostat. The Particle Mesh Ewald (54) was used for calculating long-run electrostatic interactions. A cut-off of 9 Å was used for nonbonded interactions, and the SHAKE algorithm (55) was used to restrain hydrogen atoms. All equilibrated systems were subjected to a single 1 μs long production MD run at NPT using *pmemd*.*cuda* from Amber24. Coordinates were saved every 100 ps to achieve 10,000 frames per μs of MD simulation. Convergence of each simulation was evaluated by monitoring the RMSD of the enzyme backbone atoms over the production run. All trajectories were analyzed using the *cpptraj* module of Amber25. Furthermore, calculations of features such as hydrogen bonds and atomic distances were performed using VMD. The binding energetics of the acceptor glycans were calculated using MMPBSA.py with the single-trajectory MM/GBSA protocol (igb = 2, saltcon = 0.150; default radii) over 1000 snapshots evenly spaced between 1 and 1000 ns per replicate. The RMSF of the protein residues was calculated using *cpptraj* (56). Representative structures were rendered in VMD (57) using the Symbol Nomenclature for Glycans (SNFG).

### Western and lectin blotting, CBB staining, and silver staining

Cells were sonicated in lysis buffer [TBS containing 0.5% Nonidet P-40 (NP-40) and a protease inhibitor cocktail (Fujifilm)]. Protein concentrations of the lysates were measured using Pierce BCA Protein Assay (Thermo Fisher Scientific). The cell lysates or purified proteins were mixed with Laemmli sample buffer and boiled, followed by 5 to 20% or 3 to 10% SDS-PAGE and subsequent western and lectin blotting. For CBB staining, SDS-PAGE gel was stained with GelCode Blue Safe Protein Stain (Thermo Fisher Scientific). Silver staining was performed using the Silver Staining II Kit Wako (Fujifilm) according to the attached protocol. For western blotting, proteins separated by SDS-PAGE were transferred to a nitrocellulose membrane. After blocking with TBS containing 5% skim milk and 0.1% Tween-20, the membranes were incubated with the primary antibodies overnight at 4 °C. After washing with TBS containing 0.1% Tween-20 (TBS-T), the membranes were incubated with the HRP-conjugated secondary antibodies at room temperature. After washing with TBS-T and TBS, signals were detected with Western Lightning Plus-ECL (PerkinElmer) or SuperSignal West Femto Maximum Sensitivity substrate (Thermo Fisher Scientific). For lectin blotting, nitrocellulose membranes were blocked with 2% BSA/TBS-T for HRP-L4-PHA or with TBS-T for LEL, followed by incubation with biotinylated lectin overnight at 4 °C. After washing with TBS-T, the membranes were incubated with HRP-L4-PHA or HRP-streptavidin (VECTASTAIN ABC Standard Kit). After washing with TBS-T and TBS, the protein bands were detected, as in the case of Western blotting. Images were taken using FUSION-SOLO 7s EDGE (Vilber-Lourmat). The signal intensity of the detected bands was quantified using ImageJ.

### Laminin overlay assay

Fc-tagged α−DG was purified from the media as described below (see “*Preparation of secreted Fc-tagged proteins from culture medium*” section). Purified α−DG was separated by 5 to 20% SDS-PAGE and then transferred to a nitrocellulose membrane. The membranes were blocked with TBS containing 5% skim milk, 1 mM CaCl_2_, and 1 mM MgCl_2_ at room temperature for 1 h and washed with TBS containing 1 mM CaCl_2_ and 1 mM MgCl_2_. The membrane was incubated with Laminin (mouse Engelbreth-Holm-Swarm [EHS] Tumor [120–05751, Fujifilm]) diluted with TBS containing 1 mM CaCl_2_, 1 mM MgCl_2_, and 2% BSA overnight at 4 °C. The membrane was washed and incubated with anti-laminin at room temperature for 1 h, and the signal was detected after 1 h of incubation with an HRP-conjugated secondary antibody in the same way as for the western blotting. TBS containing 1 mM CaCl_2_ and 1 mM MgCl_2_ was used at all washing steps.

### Chemical synthesis of GnM-S-Flu

M1 disaccharyl serine S1 was prepared using a previously reported procedure (22). S1 (2.9 mg, 6.2 μmol) was dissolved in H_2_O (1.2 ml) and to the solution were added 5-carboxyfluorescein *N*-succinimidyl ester (6.5 mg, 12 μmol) and trimethylamine (0.20 M solution in DMF: 0.25 ml, 50 μmol) at room temperature. After stirring at the same temperature for 26 h, the mixture was purified by size-exclusion chromatography on Sephadex LH-20 (MeOH/H_2_O = 1:1) to give 1 (2.0 mg, 39%) as an orange solid: ^1^H NMR (500 MHz, CD_3_OD) δ 8.53 (s, 1 H, Ph), 8.26 (dd, 1 H, *J* = 1.5 Hz, *J* = 8.0 Hz, Ph), 7.30 (d, 1 H, *J* = 8.0 Hz, Ph), 6.70–6.62 (m, 4 H, Ph), 6.55 (dd, 2 H, *J* = 2.2 Hz, *J* = 8.8 Hz, Ph), 4.68–3.42 (m, 17 H, H-1^*GlcNAc*^, H-2^*GlcNAc*^, H-3^*GlcNAc*^, H-4^*GlcNAc*^, H-5^*GlcNAc*^, H-6a^*GlcNAc*^, H-6b^*GlcNAc*^, H-1^*Man*^, H-2^*Man*^, H-3^*Man*^, H-4^*Man*^, H-5^*Man*^, H-6a^*Man*^, H-6b^*Man*^, H-α^*Ser*^, H-βa^*Ser*^, H-βb^*Ser*^), 2.67 (s, 3 H, Ac); MS (MALDI) *m/z*: found [M − H]^−^ 827.38, C_38_H_40_N_2_O_19_ calcd for [M − H]^−^ 827.22.

### MS analysis

MALDI-TOF MS spectra were acquired on a Bruker microflex mass spectrometer (Bruker Daltonics, Germany) using α-cyano-4-hydroxycinnamic acid (20 mg/ml in 50:50 MeCN/0.1% TFA, v/v) as the matrix. ESI-TOF MS spectra in negative ion mode were acquired on a Bruker micrOTOF mass spectrometer.

### NMR

^1^H NMR spectra were recorded on an AVANCE III 500 MHz spectrometer (Bruker). Chemical shifts in ^1^H NMR spectra are reported in ppm (δ) relative to tetramethylsilane (TMS, δ = 0.00). Data are given in the following order: chemical shift, multiplicity (s = singlet, d = doublet, dd = double doublet, m = multiplet), coupling constant(s) in Hz, and integration.

### Purification of recombinant enzymes

Purification of recombinant 6 × His-tagged soluble enzymes was carried out as described previously (58). In brief, 60 to 80% confluent COS7 or HEK293 T cells on 15-cm dishes were transfected with the plasmids using polyethylenimine MAX. After 6 h of transfection, the culture medium was replaced with Opti-MEM I, followed by further culture for 48 to 72 h at 37 °C. The culture medium was collected, and the cell debris was removed by centrifugation. Then, 6 × His-tagged enzymes were purified using a Ni^2+^-column and desalted using a NAP-5 gel filtration column (Cytiva).

### Assays of glycosyltransferase and sulfotransferase activity

To measure the activity of GnT-IX and GnT-V toward *N*-glycans, purified enzymes were incubated in 10 μl of reaction buffer (125 mM MES, pH 6.25, 10 mM EDTA, 0.5% TritonX-100, 1 mg/ml BSA) containing 20 mM UDP-GlcNAc and 10 μM fluorescently labeled acceptor substrate (GnGnbi-PA) at 37 °C for 16 h or 1 h, as described previously (21). The reaction mixture was boiled at 99 °C for 2 min to stop the reaction, followed by the addition of 40 μl of water. The mixture was centrifuged at 21,500×*g* for 5 min, and 10 μl of the supernatant was injected into an HPLC system equipped with an ODS column (Inertsil ODS-3, 4.6 × 250 mm; GL Sciences) to detect the fluorescence-conjugated acceptor substrate and the products. The mobile phase consisted of 80% solvent A (20 mM acetate buffer, pH 4.0 adjusted by aqueous ammonia) and 20% solvent B (solvent A containing 1% 1-butanol).

To measure the activity toward *O*-Man glycan, purified GnT-IX was incubated in 10 μl of reaction buffer (50 mM HEPES, pH 7.5, 10 mM MnCl_2_, 0.5% TritonX-100, 1 mg/ml BSA) containing 20 mM UDP-GlcNAc and 10 μM fluorescently labeled acceptor substrate (GnM-S-Flu) at 37 °C for 3 h. To measure the activity of GnT-V toward the same acceptor substrate, GnT-V was incubated in 10 μl of reaction buffer (125 mM MES, pH 6.25, 10 mM EDTA, 0.5% TritonX-100, 1 mg/ml BSA) containing 20 mM UDP-GlcNAc and 10 μM GnM-S-Flu at 37 °C for 1 h. The reaction mixtures were boiled at 99 °C for 2 min to stop the reactions, followed by the addition of 40 μl of water. The mixtures were centrifuged at 21,500 x g for 5 min, and 10 μl of the supernatants was injected into an HPLC system equipped with an ODS column (Inertsil ODS-3, 4.6 × 250 mm; GL Sciences) to detect the fluorescence-conjugated acceptor substrate and the product. The mobile phase consisted of 80% solvent A (20 mM acetate buffer, pH 4.0 adjusted by aqueous ammonia) and 20% solvent B (100% acetonitrile).

To measure the activity of B4GALT1, B4GALT4, B3GNT7, CHST1, CHST2 and CHST6, toward the linear or branched O-Man glycans, purified enzyme was incubated in 10 μl of reaction buffer (50 mM MES, pH 6.25, 10 mM MnCl2, 0.5% TritonX-100, 1 mg/ml BSA) containing donor substrate (20 mM UDP-GlcNAc for B3GNT7, 10 mM UDP-Gal for B4GALT1 and B4GALT4, or 1 mM PAPS for CHSTs) and 10 μM fluorescently labeled acceptor substrates (GnM-S-Flu [G1] and Gn(Gn)M-S-Flu [G4] for B4GALT1, B4GALT4; GalGnM-S-Flu [G2] and GalGn(GalGn)M-S-Flu for B3GNT7 [G5]; GnGalGnM-S-Flu [G3], and GnGalGn(GnGalGn)M-S-Flu [G6] for CHSTs) at 37 °C. The reaction mixtures were boiled at 99 °C for 2 min to stop the reactions, followed by the addition of 40 μl of water. The mixtures were centrifuged at 21,500×*g* for 5 min, and 10 μl of the supernatants was injected into an HPLC system equipped with an ODS column (Inertsil ODS-3, 4.6 × 250 mm; GL Sciences) to detect the fluorescence-conjugated acceptor substrates and the products. The mobile phase consisted of 80% solvent A (20 mM acetate buffer, pH 4.0 adjusted by aqueous ammonia) and 20% solvent B (100% acetonitrile) for B4GALT1, B4GALT4 and B3GNT7, and 80% solvent A (20 mM acetate buffer with 5 μM citric acid, pH 4.0 adjusted by aqueous ammonia) and 20% solvent B (100% acetonitrile) for CHST1, CHST2, and CHST6.

To enzymatically synthesize Gn(Gn)M-S-Flu, purified GnT-V T288 R was incubated in reaction buffer (50 mM MES, pH 6.25, 10 mM MnCl_2_, 0.5% TritonX-100, 1 mg/ml BSA) containing 40 mM UDP-GlcNAc and the fluorescently labeled acceptor substrate GnM-S-Flu at 37 °C overnight. To synthesize GalGnM-S-Flu, GalGn(GalGn)M-S-Flu, GnGalGnM-S-Flu, and GnGalGn(GnGalGn)M-S-Flu, purified B4GALT1 (for GalGnM-S-Flu and GalGn(GalGn)M-S-Flu), or B3GNT7 (for GnGalGnM-S-Flu and GnGalGn(GnGalGn)M-S-Flu), were incubated in reaction buffer (50 mM MES, pH 6.25, 10 mM MnCl2, 0.5% TritonX-100, 1 mg/ml BSA) containing donor substrate (25 mM UDP-Gal for B4GALT1 and 40 mM UDP-GlcNAc for B3GNT7) and fluorescently labeled acceptor substrates (615 μM GnM-S-Flu and 685 μM Gn(Gn)M-S-Flu for B4GALT1; 392 μM GalGnM-S-Flu and 349 μM GalGn(GalGn)M-S-Flu for B3GNT7) at 37 °C overnight. The reaction mixtures were boiled at 99 °C for 2 min to stop the reactions, followed by the addition of 40 μl of water. The mixtures were then centrifuged at 21,500×*g* for 5 min, and 10 μl of the supernatants were injected into an HPLC system equipped with an ODS column (Inertsil ODS-3, 4.6 × 250 mm; GL Sciences) to separate the fluorescence-conjugated acceptors and the products and collect these products. The mobile phase consisted of 80% solvent A (20 mM acetate buffer, pH 4.0 adjusted by aqueous ammonia) and 20% solvent B (100% acetonitrile), and the products were purified. After drying the products using CentriVap (LABCONCO), the concentration was adjusted to 100 μM and the purified glycans were used for the enzyme assays as acceptor substrates.

### Kinetic analysis of B4GALT1

To determine the kinetic parameters of B4GALT1 toward the linear or branched *O*-Man glycans, the purified enzyme was incubated in 10 μl of reaction buffer (50 mM MES, pH 6.25, 10 mM MnCl_2_, 0.5% TritonX-100, 1 mg/ml BSA) containing 10 mM UDP-Gal and various concentrations of the fluorescently labeled acceptor substrates (GnM-S-Flu (227.4, 454.7, 909.4, 1818, 2728, and 3637 μM) and Gn(Gn)M-S-Flu (5.030, 10.06, 20.12, 40.24, 80.49, 161.0, and 322.0 μM) at 37 °C. The reaction mixtures were boiled at 99 °C for 2 min to stop the reactions, followed by the addition of 40 μl of water. The mixtures were centrifuged at 21,500×*g* for 5 min, and 10 μl of the supernatants was injected into an HPLC system equipped with an ODS column (Inertsil ODS-3, 4.6 × 250 mm; GL Sciences) to detect the fluorescence-conjugated acceptor substrates and the products. The mobile phase consisted of 80% solvent A (20 mM acetate buffer, pH 4.0 adjusted by aqueous ammonia) and 20% solvent B (100% acetonitrile). The kinetic parameters were calculated using GraphPad Prism 8.

### Preparation of secreted Fc-tagged proteins from culture medium

After 4 h of transfection, the culture medium was replaced with Opti-MEM I, followed by a further 48 h of culture. The medium was collected and centrifuged at 1200 x *g* for 5 min to remove cell debris, and the supernatants were incubated with Dynabeads protein G (Thermo Fisher Scientific), which had been preequilibrated with PBS, at 4 °C overnight. The beads were washed three times with PBS and then subjected to laminin overlay assay (α−DG-Fc) or treatments with various glycosidases as described below (phosphacan-Fc).

### Animal experiments

GnT-IX-KO (*Mgat5b*^*−/−*^) mice and POMGNT1-KO (*Pomgnt1*^−/−^) mice were generated as described in previous reports (4, 59). Mice with the C57BL/6N genetic background were housed at four or fewer per cage and bred at 20∼26 °C and 40∼70% humidity, under a cycle of 12 h light/12 h dark. All experiments followed the guideline for animal experiments of Gifu University and approved by the institutional Animal Experiment Committee of Gifu University (approved number: AG-P-C-20250025).

### Preparation of brain soluble fraction

GnT-IX (*Mgat5b*) heterozygous, GnT-IX-KO, WT, and POMGNT1-KO mouse brains were homogenized in TBS containing protease inhibitor cocktail using a Potter homogenizer. The homogenates were centrifuged at 500×*g* for 10 min to remove nuclei and unbroken cells, and the supernatant was ultracentrifuged at 105,000×*g* for 30 min. The supernatant was used as a soluble fraction. To precipitate proteins in the soluble fraction, 1/30 volume of NaCl and 2.5 volumes of 70% ethanol were added, followed by incubation at −30 °C for 30 min. After centrifugation at 14,000 rpm (T15A39, himac) for 20 min, the pellet was washed with 1 ml of 70% ethanol. After additional centrifugation at 14,000 rpm (T15A39, himac) for 10 min, the pellet was used for glycosidase treatment.

### Treatment with ChABC, PNGaseF, sialidase, endo-β-galactosidase, and keratanase II

For ChABC treatment, samples were incubated with water or *Proteus vulgaris* ChABC (Sigma, C2905) in ChABC buffer (0.1 M Tris-HCl, pH 7.4, 30 mM NaOAc, pH 5.0, and 5 mM EDTA) at 37 °C for 2 h. For PNGaseF treatment, samples were denatured in denaturing buffer (PBS containing 1% NP-40, 0.5% SDS, 1% β-mercaptoethanol, and 20 mM EDTA) at 95 °C for 5 min, followed by 10-fold dilution with PBS containing NP-40 (final concentration of 0.75% v/v). Samples were then incubated with water or PNGaseF (NEB, P0704) at 37 °C for 2 h. The samples were then mixed with 5 × Laemmli sample buffer and incubated at 95 °C for 5 min. For sialidase treatment, *Clostridium perfringens* α2-3,6,8 sialidase (New England Biolabs, P0720) was added simultaneously with PNGaseF. For endo-β-galactosidase treatment, the ChABC-treated samples were denatured in denaturing buffer (50 mM MES, pH 6.5, containing 1% NP-40, 0.5% SDS, 1% β-mercaptoethanol, and 20 mM EDTA) at 95 °C for 5 min, followed by 10-fold dilution with 50 mM MES, pH 6.5, containing NP-40 (final concentration of 0.75% v/v). Samples were then incubated with water or *f*. *keratoyticus* endo-β-galactosidase (R&D Systems, 8620-GH) at 37 °C for 2 h. The samples were then mixed with 5 × Laemmli sample buffer and incubated at 95 °C for 5 min.

For sequential treatments with ChABC and PNGaseF, Phosphacan-Fc-bound Dynabeads protein G were washed three times with PBS after ChABC treatment, and the PNGaseF treatment was subsequently performed on the beads. For the soluble fractions from mouse brain, proteins were first precipitated with ethanol and treated with ChABC. Subsequently, the proteins were again precipitated with ethanol, and the PNGaseF treatment was performed against these precipitated proteins.

For PNGaseF, endo-β-galactosidase, and keratanase II treatments of B3GNT7, after adding EDTA to the B3GNT7 solution dissolved in 50 mM MES buffer (pH 6.25) to a final concentration of 20 mM, samples were then incubated with water, PNGaseF, endo-β-galactosidase, or keratanase II (TGI, K0069) at 37 °C for 2 h. The samples were then mixed with 5 × Laemmli sample buffer and incubated at 95 °C for 5 min.

### RNA extraction, reverse transcription, and real-time PCR

Total RNA from mouse brain was extracted using TRI Reagent (Molecular Research Center, Inc.) according to the manufacturer's protocol. One μg of total RNA was reverse-transcribed using Superscript IV (Life Technologies) with random hexamers. For real-time PCR, cDNA was mixed with TaqMan Universal PCR master mix (Life Technologies) with primer-probe mix and amplified using a CFX Connect Real-Time PCR Dectection System (Bio-Rad). The levels of mRNAs were normalized to the corresponding GAPDH levels. The primers and probes were purchased from Applied Biosystems as follows: Mm00480752_m1 for *B4galt1* Mm00480087_m1 for *B4galt4*, Mm01295423_m1 for *B3gnt7*, Mm01334076_m1 for *Chst1*, Mm00490018_g1 for *Chst2*, Mm00517342_m1 for *Chst5*, and 4,308,313 (TaqMan Rodent GAPDH Control Reagents) for *Gapdh*.

### Statistical analysis

Statistical analysis was performed using GraphPad Prism 8 (GraphPad Software). n indicates biological replicates, and the tests used were described in the Figure legends.

## Data availability

All data are contained within this manuscript.

## Supporting information

This article contains supporting information.

## Conflict of interest

The author is an Editorial Board Member/Editor-in-Chief/Associate Editor/Guest Editor for this journal and was not involved in the editorial review or the decision to publish this article.

One of the authors (Y. K.) is an Editorial Board Member for this journal but was not involved in the editorial review or the decision to publish this article.

The authors declare that they do not have any conflicts of interest with the content of this article.
