# Supplementary material for: Enzymatic basis of branching and extension of O-Man glycans for keratan sulfate biosynthesis
Source: J Biol Chem. 2026 Jan 7;302(2):111140. doi: 10.1016/j.jbc.2026.111140 (PMC12860957; doi:10.1016/j.jbc.2026.111140)
Supplement: Supplementary Material [file mmc1.pdf]

Supporting Information

Enzymatic basis of branching and extension of *O*-Man glycans for keratan sulfate biosynthesis

Tomoya Itoh, Hide-Nori Tanaka, Mohit Pareek, Masamichi Nagae, Hiroshi Manya, Akemi Ido,

Sushil K. Mishra*, Yasuhiko Kizuka*

*Correspondence: Yasuhiko Kizuka, Ph.D., kizuka.yasuhiko.k8@f.gifu-u.ac.jp., Sushil K. Mishra, Ph.D,. sushil@olemiss.edu

This Supporting information includes:

Figs. S1–S13 (included in this PDF)

Table S1-S2 (included in this PDF)

Figure S1

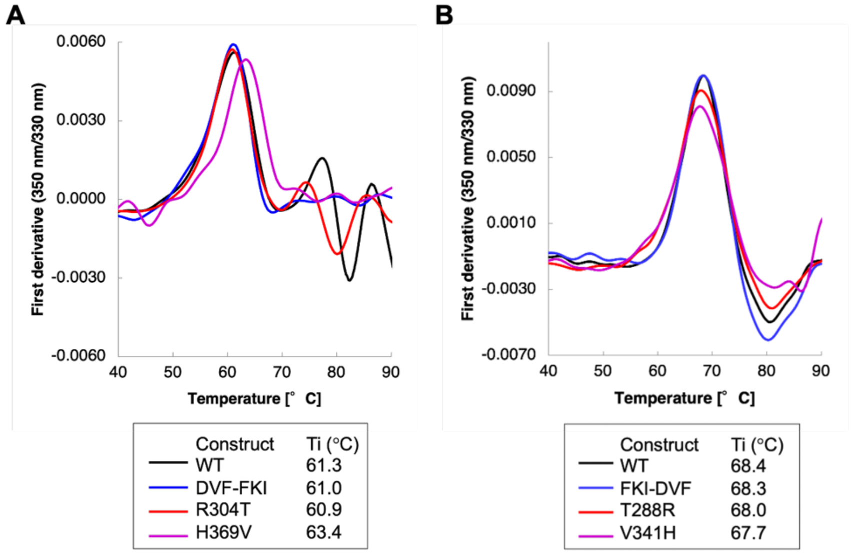

Figure S1. Thermal stability of GnT-IX and GnT-V. ***A-B*,** The first derivatives of fluorescence ratio between fluorescence at 350 and 330 nm. Soluble GnT-IX WT and the three mutants (A) and GnT-V WT and the three mutants (B) are plotted. The calculated Ti values are shown below.

Figure S2

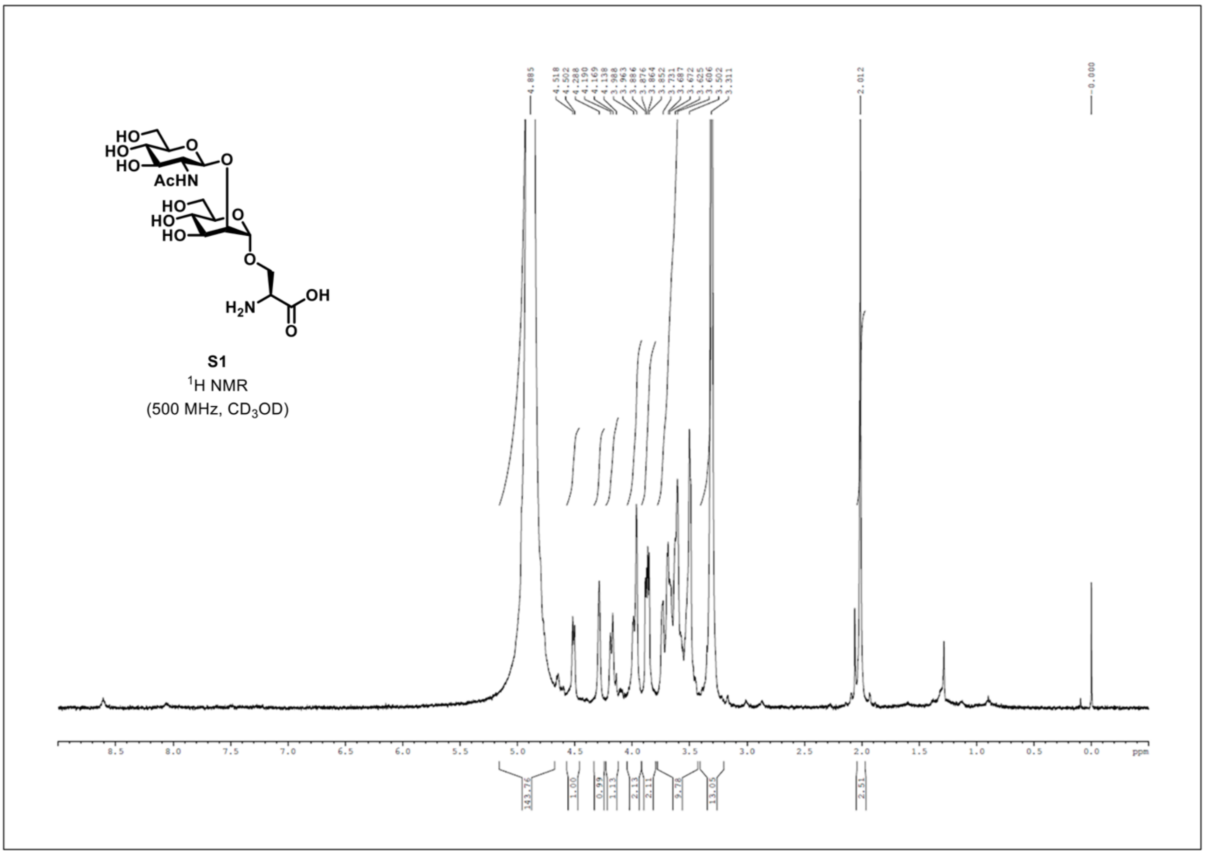

Figure S2. ^{1}H NMR spectrum of M1 disaccharyl Ser S1.

Figure S3

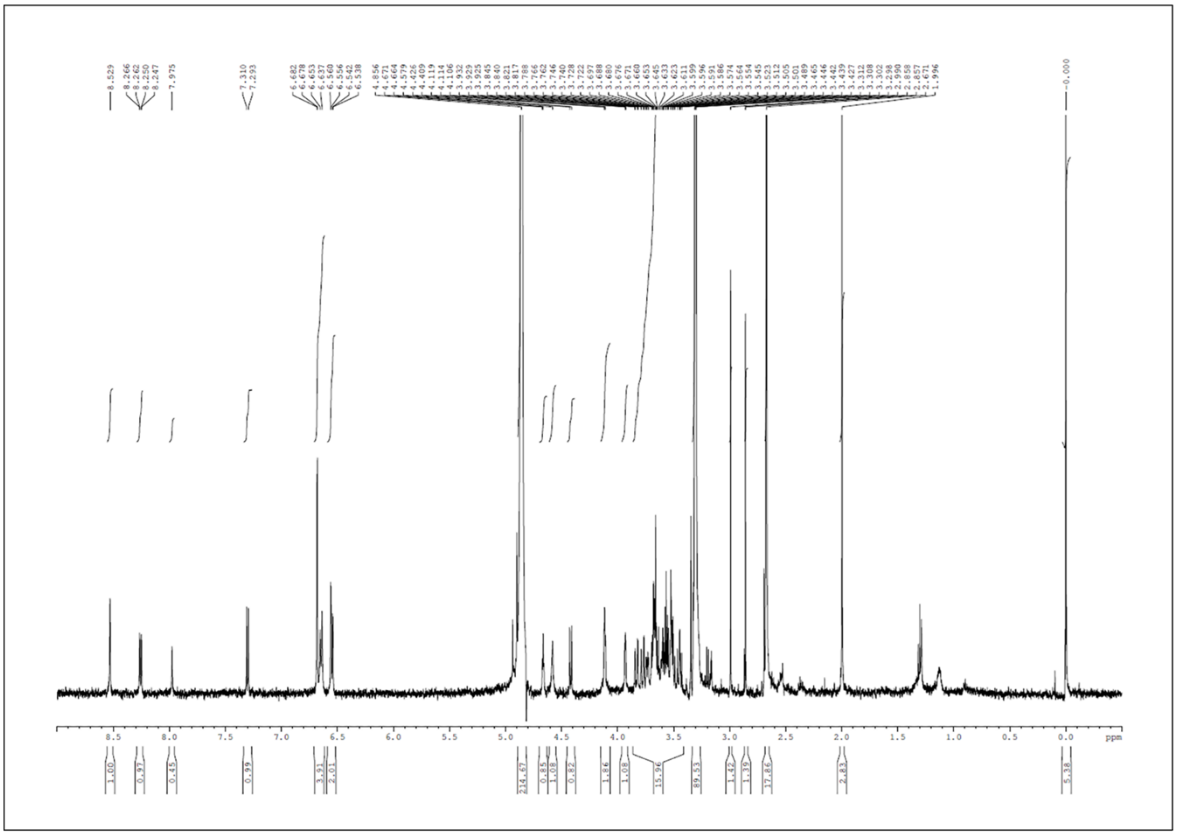

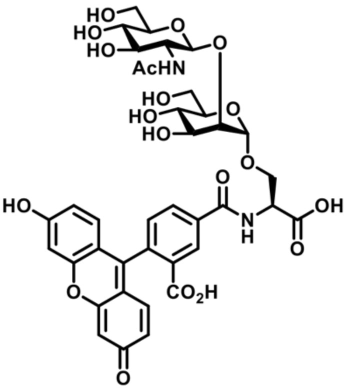

1

^{1}H NMR

(500 MHz, CD_3OD)

Figure S3. ^{1}H NMR spectrum of fluorescein-labeled M1 disaccharyl Ser S1.

Figure S4

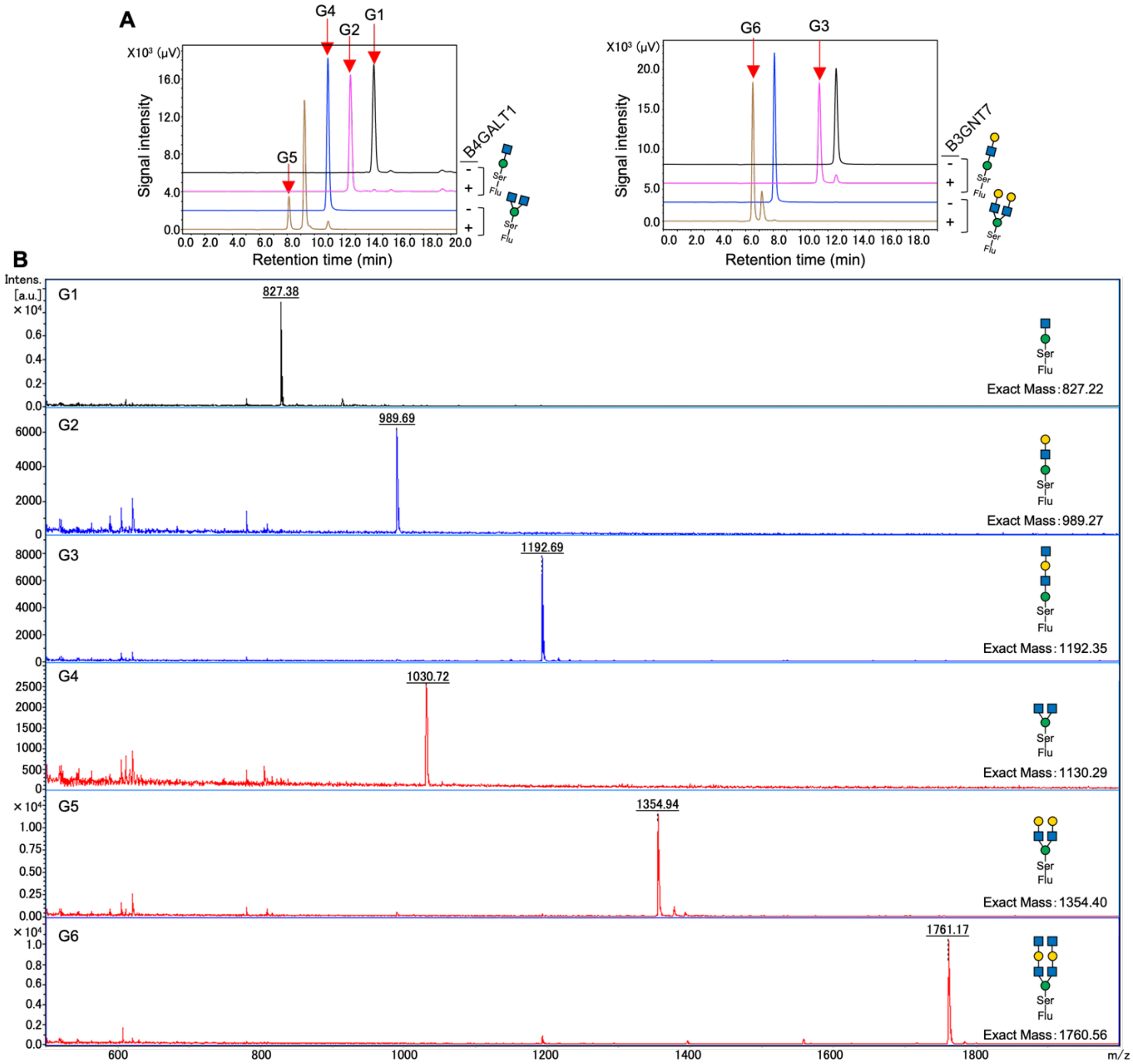

Figure S4. MALDI-TOF MS analysis of fluorescein-labeled glycans. ***A*****,** Purified B4GALT1 and B3GNT7 were incubated with GnM-S-Flu or Gn(Gn)M-S-Flu (for B4GALT1), GalGnM-S-Flu or GalGn(GalGn)M-S-Flu (for B3GNT7), and the reaction mixtures were analyzed by reverse-phase HPLC. G1: GnM-S-Flu, G2: GalGnM-S-Flu, G3: GnGalGnM-S-Flu, G4: Gn(Gn)M-S-Flu, G5: GalGn(GalGn)M-S-Flu, and G6: GnGalGn(GnGalGn)M-S-Flu. ***B*****,** MALDI-TOF MS spectra in negative ion mode were acquired on a Bruker microflex mass spectrometer (Bruker Daltonics, Germany) using α-cyano-4-hydroxycinnamic acid (20 mg/mL in 50:50 MeCN/0.1% TFA, v/v) as the matrix.

Figure S5

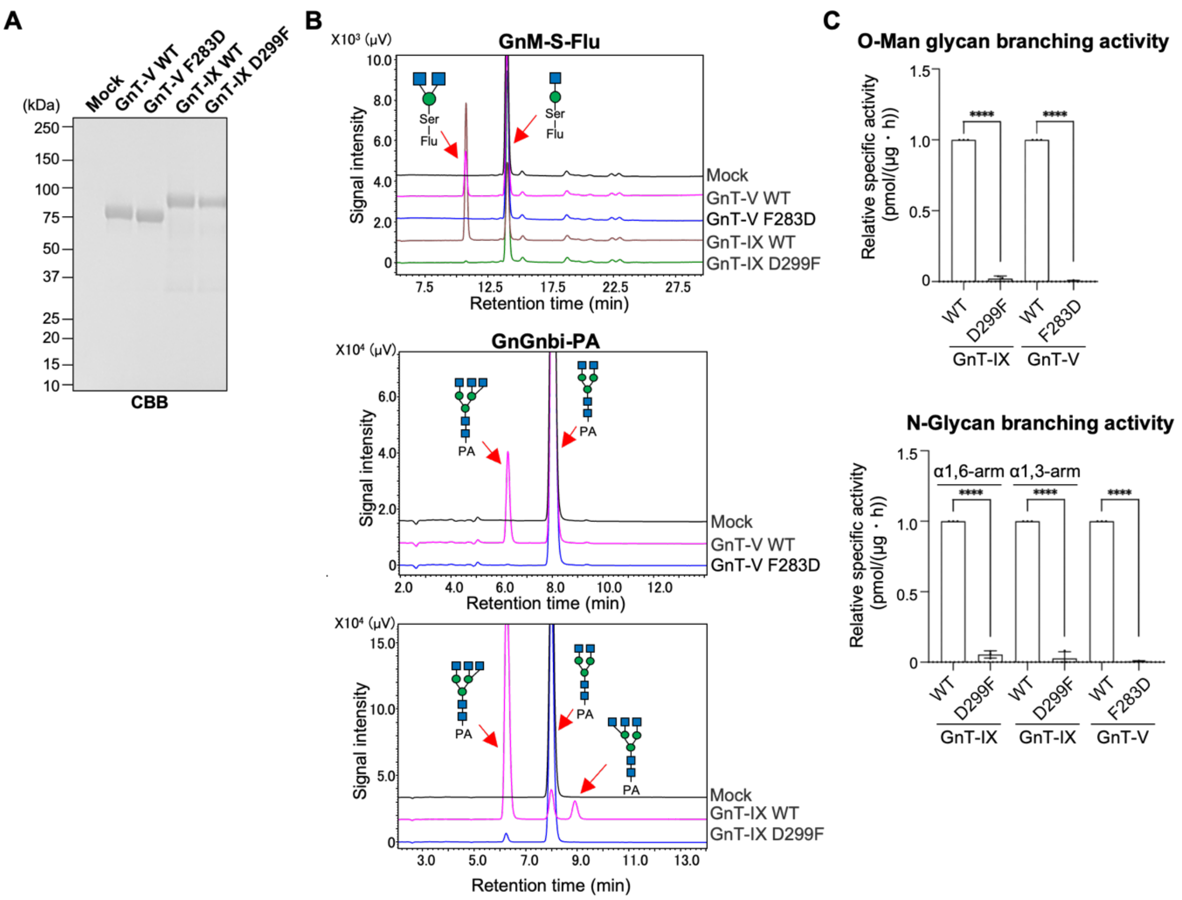

Figure S5. Purification and activity of GnT-IX D299F and GnT-V F283D. ***A,*** Soluble GnT-IX WT and D299F and GnT-V WT and F283D were expressed in HEK293T cells and purified from the medium using a Ni^{2+} column. Purified GnT-IX and -V enzymes were separated by SDS-PAGE and visualized by CBB staining. ***B,*** Purified GnT-IX WT, GnT-IX D299F, GnT-V WT, and GnT-V F283D, were incubated with GnM-S-Flu or GnGnbi-PA, and the reaction mixtures were analyzed by reverse-phase HPLC. ***C,*** The relative specific activities of GnT-IX D299F, -V F283D toward GnM-S-Flu or GnGnbi-PA were calculated from the peak areas in (B) (n = 3, mean ± SD, *****p* < 0.0001, unpaired *t* test).

Figure S6

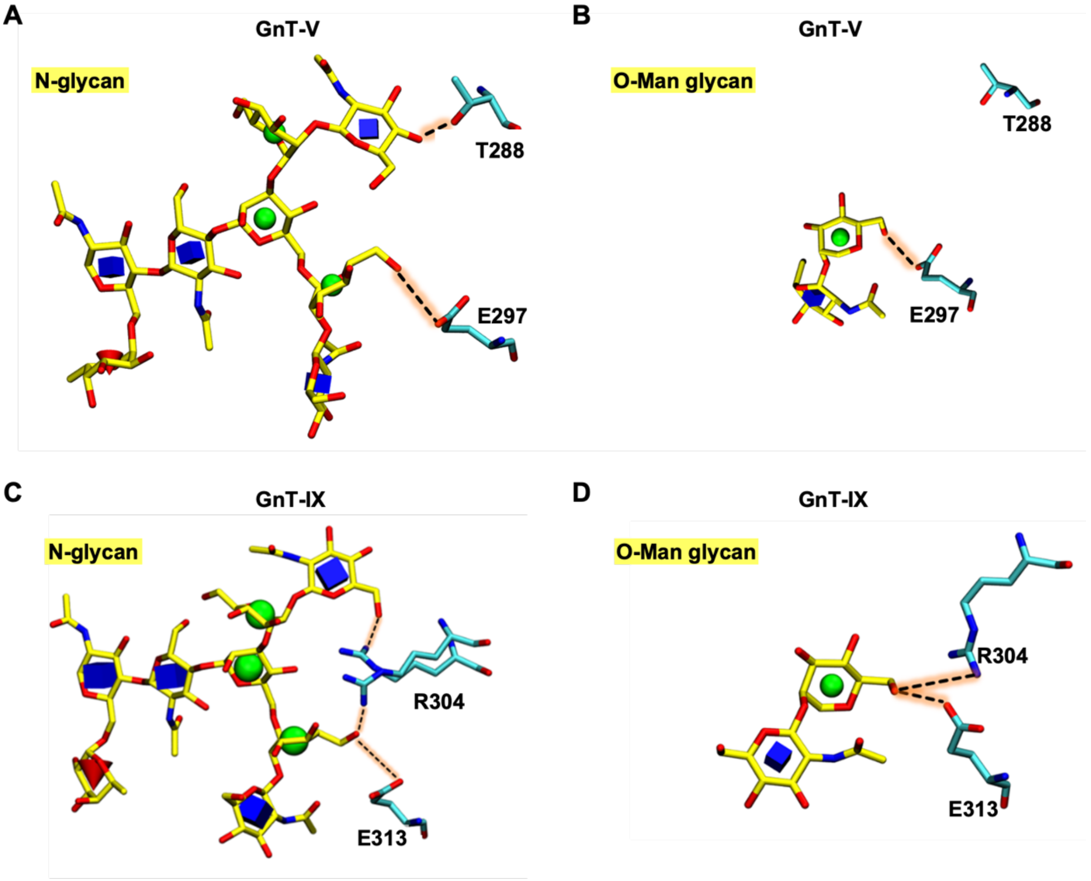

Figure S6. Graphical representation of T288 and E297 residues in GnT-V and R304 and E313 in GnT-IX in complex with acceptor substrates. ***A,*** GnT-V and *N*-glycan acceptor. ***B,*** GnT-V and *O*-Man glycan acceptor. ***C,*** GnT-IX and *N*-glycan glycan acceptor. ***D,*** GnT-IX and *O*-Man glycan acceptor.

Figure S7

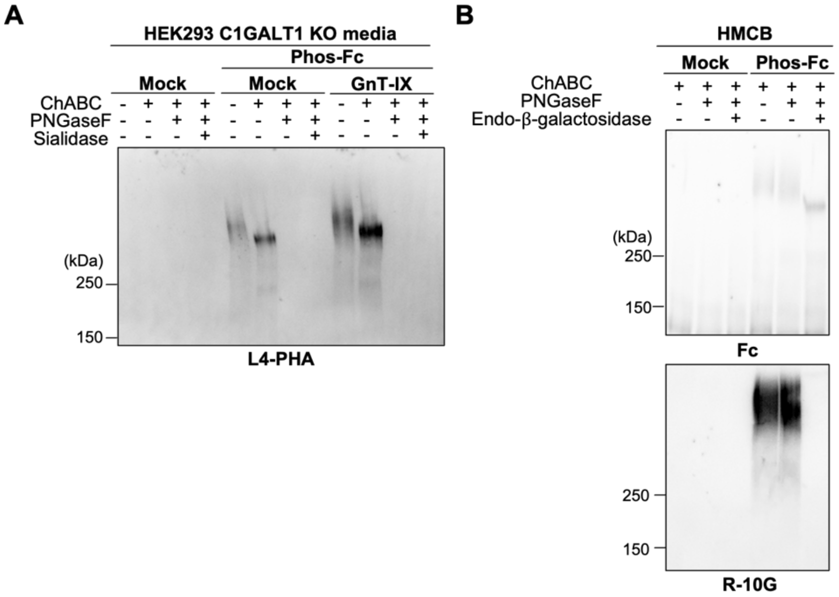

Figure S7. Analysis of *N*- and *O*-Man glycans on phosphacan in HEK293 C1GALT1-KO and HMCB cells. ***A***, Phosphacan-Fc (Phos-Fc) and GnT-IX were expressed in HEK293 C1GALT1 KO cells. Phosphacan was purified from the media using Dynabeads protein G. After treatment of phosphacan with ChABC, PNGaseF, and sialidase, proteins were separated by SDS-PAGE and blotted with L4-PHA. ***B***, Phos-Fc was expressed in HMCB cells. Phosphacan was purified from the media using Dynabeads protein G. After treatment of phosphacan with ChABC, PNGaseF, and Endo-β-galactosidase, proteins were separated by SDS-PAGE and blotted with anti-human IgG and R-10G.

Figure S8

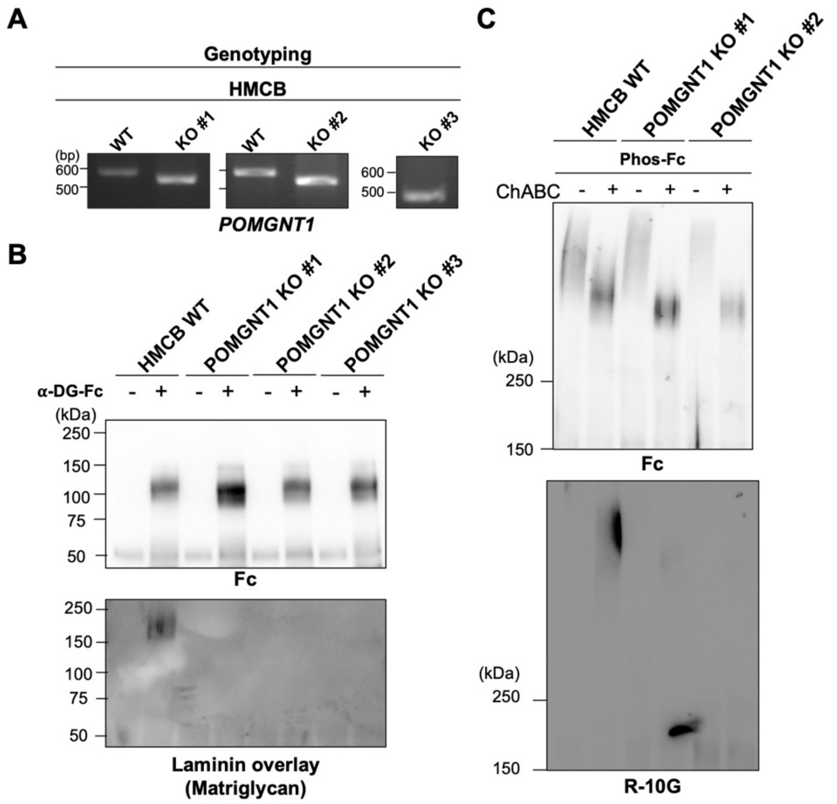

Figure S8. Generation of HMCB POMGNT1-KO cells. ***A,*** Genomic DNAs were extracted from HMCB WT or POMGNT1 KO cell clones (#1, 2, and 3), and the DNA fragments including the targeted sequence for gene editing were amplified by PCR. ***B,*** α-DG-Fc was expressed in HMCB WT and POMGNT1-KO cells and purified from the media using Dynabeads protein G. Purified α-DG-Fc was subjected to western blotting with anti-human IgG and laminin overlay assay. ***C,*** Phosphacan-Fc was expressed in HMCB WT and POMGNT1-KO cells and purified from the media using Dynabeads protein G. Purified phosphacan-Fc was treated with ChABC and was separated by SDS-PAGE and blotted with anti-human IgG and R-10G.

Figure S9

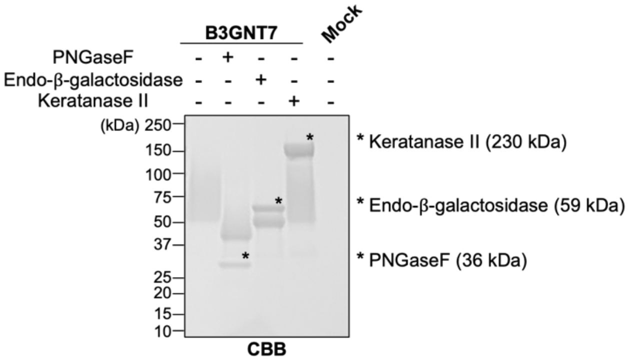

Figure S9. **Glycosidase treatment of B3GNT7.** Soluble recombinant B3GNT7 purified from HEK293T media was treated with PNGaseF, endo-β-galactosidase, or keratanase II, and then subjected to SDS-PAGE and CBB staining. The asterisks indicate the positions of glycosidases.

Figure S10

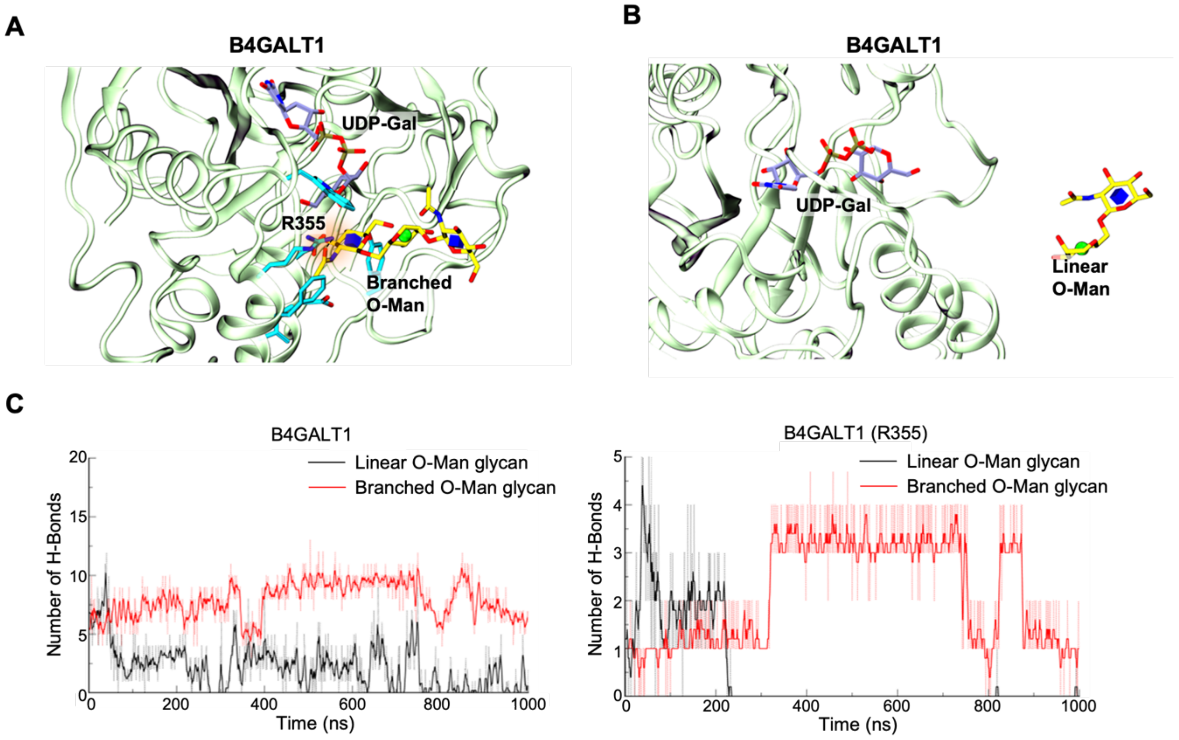

Figure S10. Binding modes of linear and branched *O*-Man glycan acceptors in B4GALT1. ***A-B***, The complex structures of B4GALT1 (PDB ID: 4EEO) with the branched (A) and linear (B) *O*-Man glycan are shown. ***C,*** The total number of hydrogen bonds formed by the linear or branched *O*-Man glycans with B4GALT1 (left) or with R355 residue (right).

Figure S11

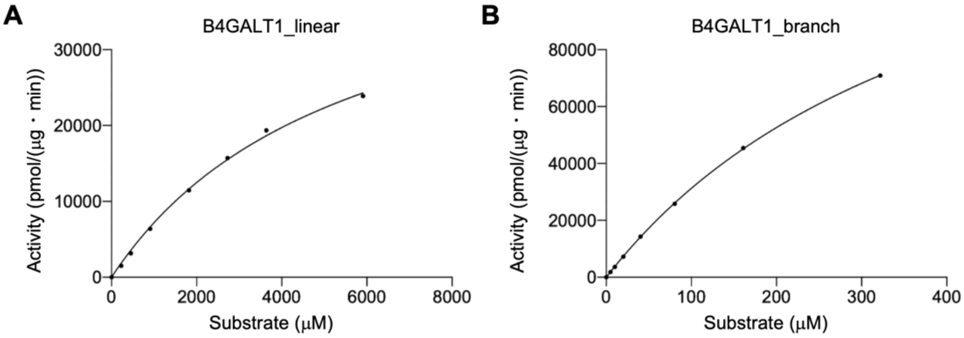

K_M (M)	5.550×10^{-3}
k_{cat} (s^{-1})	34.48
k_{cat}/K_M ($M^{-1} \cdot s^{-1}$)	6.213×10^{3}

K_M (M)	4.324×10^{-4}
k_{cat} (s^{-1})	121.8
k_{cat}/K_M ($M^{-1} \cdot s^{-1}$)	2.817×10^{5}

Figure S11. Kinetic analysis of B4GALT1 toward linear and branched *O*-Man glycans. ***A,*** B4GALT1 activity was measured under the conditions similar to Fig. 5D with various concentrations (227.4, 454.7, 909.4, 1818, 2728, 3637, 5911 μM) of GnM-S-Flu and with the enzyme (1.448×10^{-8} M). The reaction mixtures were analyzed by reversed-phase HPLC as in the case for Fig. 5D. ***B,*** B4GALT1 activity was measured under the conditions similar to Fig. 5D with various concentrations (5.030, 10.06, 20.12, 40.24, 80.49, 161.0, and 322.0 μM) of Gn(Gn)M-S-Flu and with the enzyme (1.448×10^{-8} M). The reaction mixtures were analyzed by reversed-phase HPLC as in the case for Fig. 5D. Kinetic parameters are shown in the below tables.

Figure S12

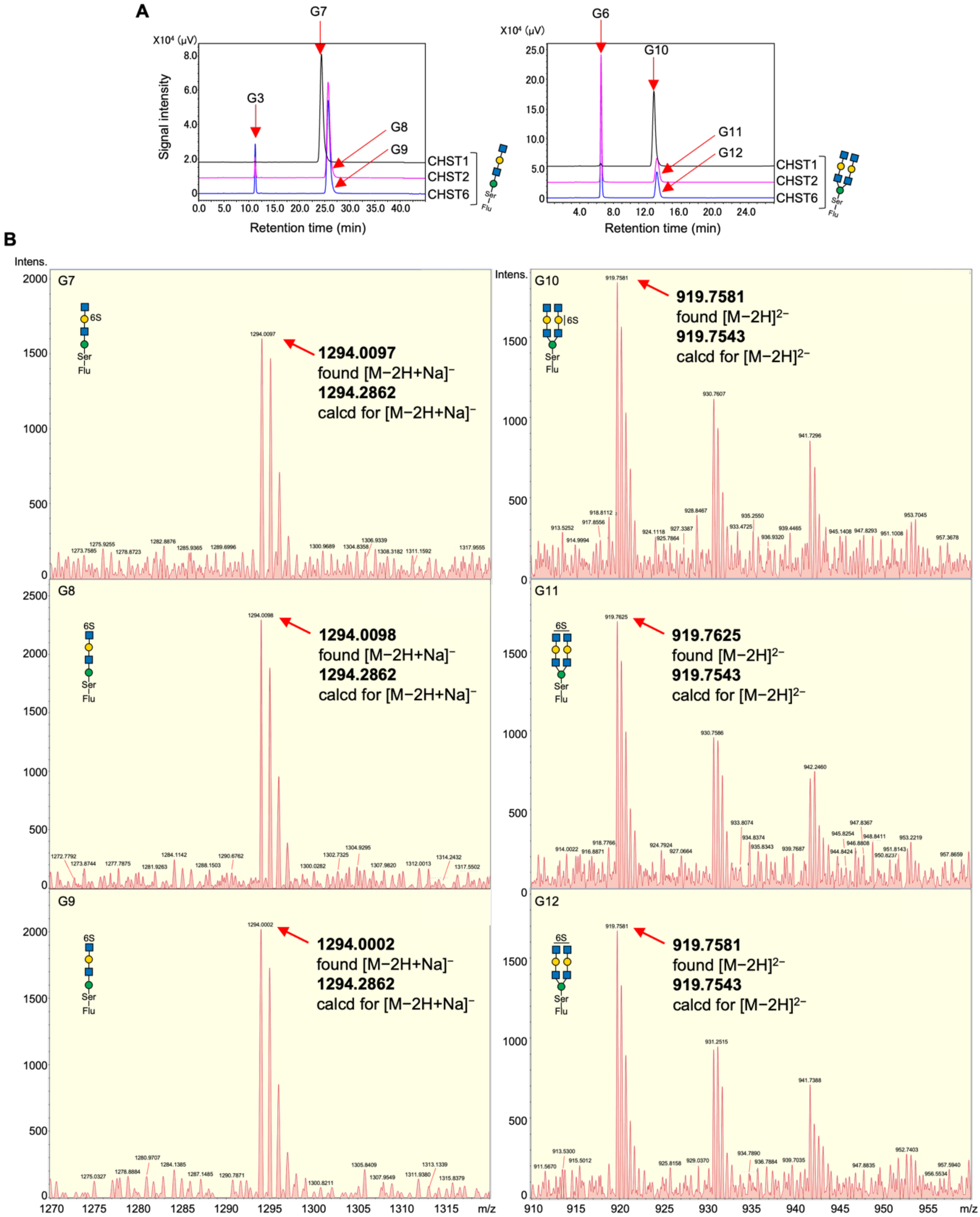

Figure S12. ESI-TOF MS analysis of fluorescein-labeled CHST products. ***A*,** Purified CHST1, CHST2, and CHST6 were incubated with GnGalGnM-S-Flu or GnGalGn(GnGalGn)M-S-Flu, and the reaction mixtures were analyzed by reverse-phase HPLC. The enzymatic products were collected and analyzed by MS. ***B*,** ESI-TOF MS spectra in negative ion mode were acquired on a Bruker micrOTOF mass spectrometer. G7, CHST1 product of GnGalGnM-S-Flu. G8, CHST2 product of GnGalGnM-S-Flu. G9, CHST6 product of GnGalGnM-S-Flu. G10, CHST1 product of GnGalGn(GnGalGn)M-S-Flu. G11, CHST2 product of GnGalGn(GnGalGn)M-S-Flu. G12, CHST6 product of GnGalGn(GnGalGn)M-S-Flu.

Figure S13

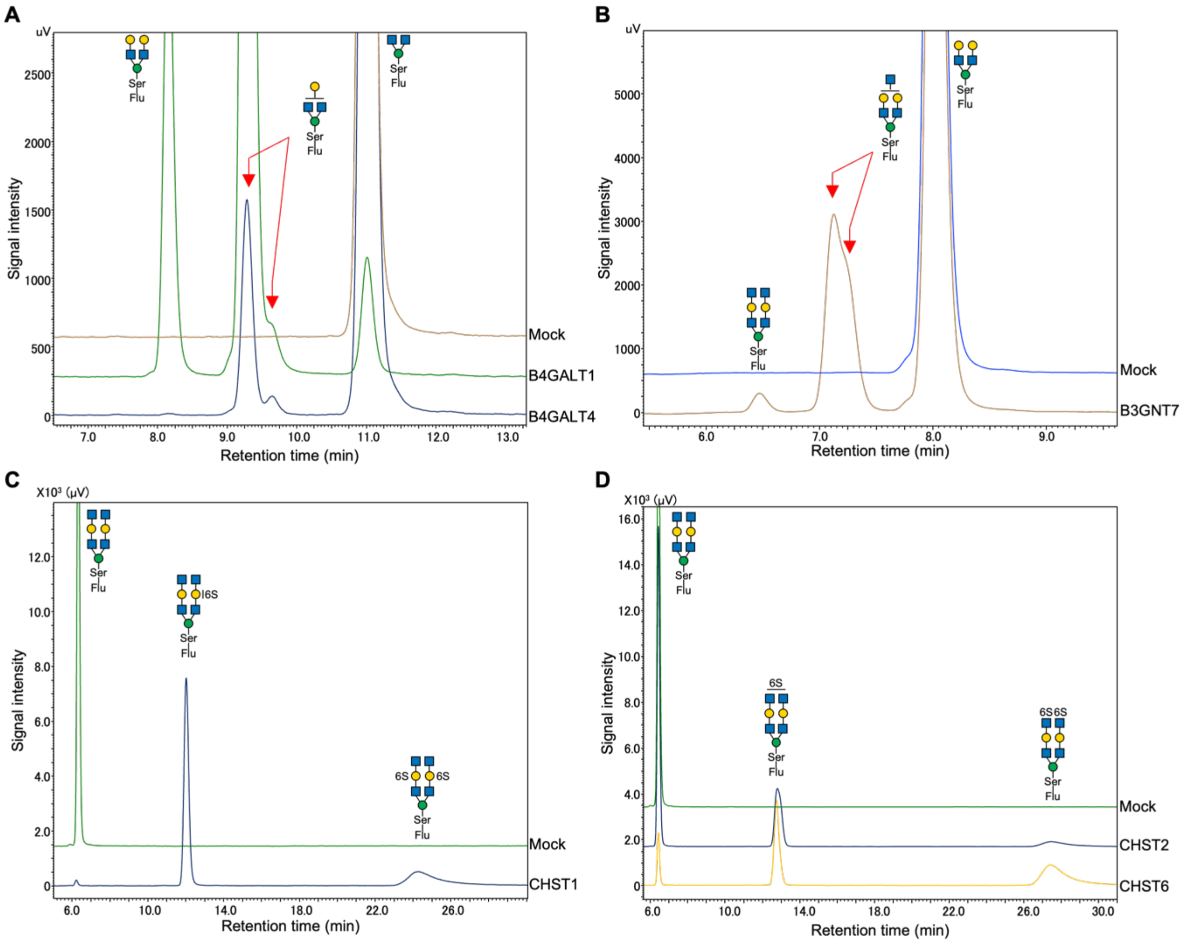

Figure S13. Activity of KS biosynthetic enzymes toward branched *O*-Man glycans. ***A-D*,** Purified B4GALT1, B4GALT4, B3GNT7, CHST1, CHST2, and CHST6 were incubated with Gn(Gn)M-S-Flu (for B4GALT1 and B4GALT4) (A), GalGn(GalGn)M-S-Flu (for B3GNT7) (B), or GnGalGn(GnGalGn)M-S-Flu (for CHSTs) (C and D), and the reaction mixtures were analyzed by reverse-phase HPLC.

Table S1.

Hydrogen-bonding between GnT-IX/GnT-V and their *N*-glycan and *O*-Man substrates.

GnT–IX/N-Glycan			GnT–V/N-Glycan		
**Residue**	**Monosaccharide**	**Occupancy**	**Residue**	**Monosaccharide**	**Occupancy**
K375	Fuc	99.1	D365	GlcNAc4	200.5
T394	GlcNAc3	62.6	S366	GlcNAc4	86.0
E313	Man2	59.7	G292	GlcNAc3	38.2
Q372	Fuc	75.6	K541	GlcNAc4	26.2
G417	Fuc	85.8	F283	GlcNAc3	24.4
D393	GlcNAc3	84.3	K347	Fuc	21.4
F395	GlcNAc3	50.7	T288	GlcNAc3	20.1
T396	GlcNAc3	34.0			
K572	GlcNAc3	31.9			
R304	Man2	26.3			

GnT–IX/O-Man Complex			GnT–V/O-Man Complex		
**Residue**	**Monosaccharide**	**Occupancy**	**Residue**	**Monosaccharide**	**Occupancy**
D393	GlcNAc	174.93	S366	GlcNAc	54.35
F395	GlcNAc	67.53	D365	GlcNAc	197.9
E313	Man	91.01	K541	GlcNAc	31.47
G571	GlcNAc	25.47	K541	Man	38.56
R304	GlcNAc	32.77			

Time-averaged hydrogen-bond occupancies between protein residues and individual monosaccharides of the bound *N*-glycan (top) and O-mannose glycan (bottom) are listed for GnT-IX (left) and GnT-V (right) from 1 µs MD simulations. For each donor–acceptor pair, the occupancy (%) reports the average number of hydrogen bonds per frame over the trajectory. Occupancies >100% indicate that, on average, more than one hydrogen bond is formed simultaneously between the indicated residue and monosaccharide. Interacting protein residue-monosaccharide pairs showing less than 15% occupancy have been omitted for clarity.

Table S2.

Primers used in this study

Oligonucleotide name	Sequence
pcDNA-IHT_For	ACATCACCATCACCATCACGAGAACCTGTACTTCCAGGGAGAATTCTGCAGATATCCAGCA
pcDNA-IHT_Rev	TGCTGGATATCTGCAGAATTCTCCCTGGAAGTACAGGTTCTCGTGATGGTGATGGTGATGT
pcDNA-IHT-IXinamori(R153-)_For	GAGAACCTGTACTTCCAGGGAGAATTCCGGCGGGACCAGTGTGAGGC
pcDNA-IHT-IXinamori(R153-)_Rev	GAGCGGCCGCCACTGTGCTGGATATCTCACAGACAGCCCTGGCACA
GnT-IX_D299F_For	TCCTGACGGAGGAGTCCGGGTTCGTGTTCAGCCCTCGGGTCCTGAAGGG
GnT-IX_D299F_Rev	CCCTTCAGGACCCGAGGGCTGAACACGAACCCGGACTCCTCCGTCAGGA
GnT-IX_DVF-FKI_For	TCCTGACGGAGGAGTCCGGGTTCAAGATCAGCCCTCGGGTCCTGAAGGG
GnT-IX_DVF-FKI_Rev	CCCTTCAGGACCCGAGGGCTGATCTTGAACCCGGACTCCTCCGTCAGGA
GnT-IX_R304T_For	CCGGGGACGTGTTCAGCCCTACGGTCCTGAAGGGCGGGCCCCT
GnT-IX_R304T_Rev	AGGGGCCCGCCCTTCAGGACCGTAGGGCTGAACACGTCCCCGG
GnT-IX_H369V_For	ACCTCATCTACACCGACTACGTCGGCCTGCAGCAGATGAAGCG
GnT-IX_H369V_Rev	CGCTTCATCTGCTGCAGGCCGACGTAGTCGGTGTAGATGAGGT
GnT-V_F283D_For	TCCTGACCAAGGAATCTGGAGATAAGATTGCAGAGACAGCTTTCAGTGG
GnT-V_F283D_Rev	CCACTGAAAGCTGTCTCTGCAATCTTATCTCCAGATTCCTTGGTCAGGA
GnT-V_FKI-DVF_For	TCCTGACCAAGGAATCTGGAGATGTGTTTGCAGAGACAGCTTTCAGTGG
GnT-V_FKI-DVF_Rev	CCACTGAAAGCTGTCTCTGCAAACACATCTCCAGATTCCTTGGTCAGGA
GnT-V_T288R_For	CTGGATTTAAGATTGCAGAGAGAGCTTTCAGTGGTGGCCCTCT
GnT-V_T288R_Rev	AGAGGGCCACCACTGAAAGCTCTCTCTGCAATCTTAAATCCAG
GnT-V_V341H_For	ACCGATCTGGCTGCCCAACTCACGGAGACAGAATTGTTGAGCT
GnT-V_V341H_Rev	AGCTCAACAATTCTGTCTCCGTGAGTTGGGCAGCCAGATCGGT
pCR-BluntII-TOPO/B4GALT1_For	TCCTGTAGCCCACACCCTTCTTAAAGC
pCR-BluntII-TOPO/B4GALT1_Rev	GTCCCTGGCTAATTTCAGGTCTCTTATC
pcDNA-IHT/B4GALT1(L127-)_For	GAACCTGTACTTCCAGGGAGCGCTGCCCGCCTGCCCTGAG
pcDNA-IHT/B4GALT1(L127-)_Rev	TGGACATCGGGACACCGAGCTAGGTCTAGAGGGCCCGTTTAAA
pcDNA6-mycHisA/B4GALT4_For	CCACTAGTCCAGTGTGGTGGGCCACCATGGGCTTCAACCTGACTTTCCACC
pcDNA6-mycHisA/B4GALT4_Rev	TGTTCGAAGGGCCCTCTAGATGCACCAAACCAGAAATCCACTGTG
pcDNA-IHT/B4GALT4(L74-)_For	GAACCTGTACTTCCAGGGAGAACTTGACAACTGCCCTTCT
pcDNA-IHT/B4GALT4(L74-)_Rev	ATTTCTGGTTTGGTGCATGAGTCTAGAGGGCCCGTTTAAA
pcDNA6-mycHisA/B3GNT7_For	CCACTAGTCCAGTGTGGTGGGCCACCATGTCGCTGTGGAAGAAAACCGTCT
pcDNA6-mycHisA/B3GNT7_Rev	TGTTCGAAGGGCCCTCTAGAGAGCACCTGGAGCTTGCGGG
pcDNA-IH/B3GNT7(L35-)_For	GACATCACCATCACCATCACCTGCAGGAGCCTCCGCCACCCACCC
pcDNA-IH/B3GNT7(L35-)_Rev	TGCTGGATATCTGCAGAATTTCAGAGCACCTGGAGCTTGCGGGAGCAG
pcDNA6-mycHisA/CHST1_For	TCCACTAGTCCAGTGTGGTGGGCCACCATGCAATGTTCCTGGAAGGC
pcDNA6-mycHisA/CHST1_Rev	TCGAAGGGCCCTCTAGACTCGAGCGAGAAGGGGCGGAAGTCCC
pcDNA-IH/CHST1(G41-)_For	GACATCACCATCACCATCACGGGCTGGCCGAGCGACTGTGCGAGG
pcDNA-IH/CHST1(G41-)_Rev	TGCTGGATATCTGCATCACGAGAAGGGGCGGAAGTCCCGCTCCTC
pcDNA6-mycHisA/CHST2_For	CCACTAGTCCAGTGTGGTGGGCCACCATGAGCCGCAGCCCGCAGCG
pcDNA6-mycHisA/CHST2_Rev	TGTTCGAAGGGCCCTCTAGAGAGACGGGGCTTCCGAAGCAGGGTC
pcDNA-IH/CHST2(A118-)_For	GACATCACCATCACCATCACGCCCGTTTGGACCTCCGCACTCCTT
pcDNA-IH/CHST2(A118-)_Rev	TGCTGGATATCTGCAGAATTTCAGAGACGGGGCTTCCGAAGCAGGGTC
pcDNA6-mycHisA/CHST6_For	CCACTAGTCCAGTGTGGTGGGCCACCATGTGGCTGCCGCGCGTCTCCAGCA
pcDNA6-mycHisA/CHST6_Rev	TGTTCGAAGGGCCCTCTAGAATTTCGGGGGTGCGAGGCGGTGGAT
pcDNA-IH/CHST6(A35-)_For	GACATCACCATCACCATCACGCAGGCGGCGAGGCGCGCGTGCATGT
pcDNA-IH/CHST6(A35-)_Rev	TGCTGGATATCTGCAGAATTTCAATTTCGGGGGTGCGAGGCGGTGGAT
px330-puro_POMGNT1#1_For	caccGCCCTACCATAGTCTTGCTC
px330-puro_POMGNT1#1_Rev	aaacGAGCAAGACTATGGTAGGGC
px330-puro_POMGNT1#2_For	caccTGATGGCTCGCCGAGTGTCC
px330-puro_POMGNT1#2_Rev	aaacGGACACTCGGCGAGCCATCA
px330-puro_POMGNT1#3_For	caccAGTCACCAGCAGGAAAAGCA
px330-puro_POMGNT1#3_Rev	aaacTGCTTTTCCTGCTGGTGACT
